# Contrasting Biofilm-Modulating Effects of Polymeric Quaternary Ammonium Compounds on the Pathogenic Yeasts *Candida albicans*, *Candidozyma auris*, and *Candida parapsilosis*

**DOI:** 10.1021/acsomega.6c02434

**Published:** 2026-07-10

**Authors:** Andreas Schelhorn, Josef Achhammer, Denis Hirsch, Jan-Christoph Walter, Daniel Gruber, Ann-Kathrin Kissmann, Frank Rosenau, Ulrich Ziener

**Affiliations:** † Institute of Organic Chemistry III-Macromolecular Chemistry and Organic Materials, University of Ulm, Albert-Einstein-Allee 11, D-89081 Ulm, Germany; ‡ Institute of Pharmaceutical Biotechnology, 9189 Ulm University, Albert-Einstein-Allee 11, 89081 Ulm, Germany; § Faculty of Medicine and Dentistry, Danube Private University, Steiner Landstraße 124, 3500 Krems an der Donau, Austria

## Abstract

Quaternary ammonium compounds (QACs), particularly in polymeric form (polyQACs), offer a broad spectrum of antimicrobial activity, e.g., via electrostatic membrane interaction. On the other hand, adamantane derivatives are notable for their rigid, lipophilic structure and exhibit a broad spectrum of biological activities. In this multidisciplinary study, we report the synthesis of three structurally defined and related polyQACs with and without azaadamantane units differing primarily in hydrophilicity and flexibility. We demonstrate that one of these compounds, poly­(vinylbenzyltrimethylammonium chloride) **3**, displays biofilm inhibition properties against the pathogenic fungi *Candidozyma auris* and *Candida parapsilosis*, whereas the remaining compounds predominantly enhance their biofilm formation. Remarkably, all compounds potentiate the biofilm formation of *Candida albicans* by up to 838% on glass surfaces and even 26-fold on polystyrene. Despite these substantial effects on biofilm biomass, planktonic cell viability remained unaffected. A tentative model for the structure–property relation between the polyQACs and biofilm formation is proposed. Although further research is needed to fully understand the biofilm-potentiating properties of these polymers, these findings may already pave the way for new diagnostic approaches and innovative treatment strategies.

## Graphical abstract

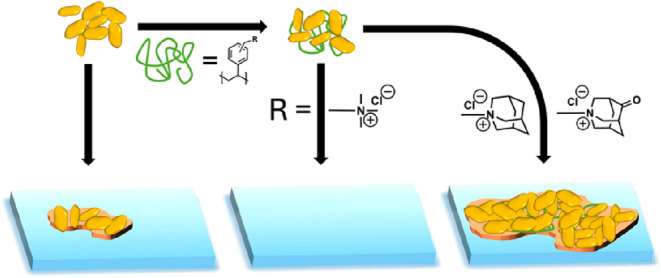

## Introduction

Prevalent fungal infections caused by *Candida* species—often conceptualized under the overarching term candidiasis—remain a major threat to the global health system.^1,2^ Among the various *Candida* species, *Candida albicans* is the predominant pathogen,^1,3,4^ but recent decades have been marked by an increasing incidence of severe infections caused by other *Candida* species.^5–8^ *Candida parapsilosis* has emerged as a pathogenic yeast of high clinical interest,^8–11^ exhibiting reduced sensitivity to conventional antifungal agents and therefore limiting the effectiveness of established therapeutic approaches.^12–15^ *Candidozyma auris* (formerly *Candida auris*) represents a relatively new but increasingly alarming pathogenic yeast species. It has developed diverse resistance mechanisms across different geographic regions, thereby limiting or even preventing the effectiveness of conventional antifungal therapies.^16–21^ *C. auris* has been classified as an emerging “superbug” issuing clinical guidance and promoting broader public health awareness and discussions.^22^ The World Health Organization (WHO) has ranked *C. parapsilosis* in the high-priority group, while *C. albicans* and *C. auris* were categorized in the group of highest concern, named the critical priority group. This prioritization underscores the urgent need for the development of novel and alternative therapeutic strategies against these pathogenic yeasts.

One of the major determinants of *C. albicans*, *C. auris*, and *C. parapsilosis* pathogenicity is its ability to form biofilms, which is widely accepted to be the predominant lifestyle of these organisms.^23–30^ Biofilms refer to structured and resilient networks that emerge when large numbers of planktonic (free-floating) cells assemble into an organized, multicellular network.^29,31–35^ In general, this complex microbial biofilm formation proceeds through four distinct phases: initial cell attachment of round yeast cells to a biotic or abiotic surface and other cells, followed by the establishment of a basal layer that provides structural anchorage for the biofilm (adherence). Aggregation, proliferation, and early growth of the yeast form cells into microcolony-like structures (growth initiation). The development of mature, species-dependent biofilm architectures, together with the formation and elongation on hyphae and pseudohyphae during biofilm growth determines the macroscopic biofilm morphology (maturation). The eventual release of cells from the biofilm matrix into the surrounding environment seeds new sites (dispersal) (Figure 1).^36–38^

**Figure 1:**
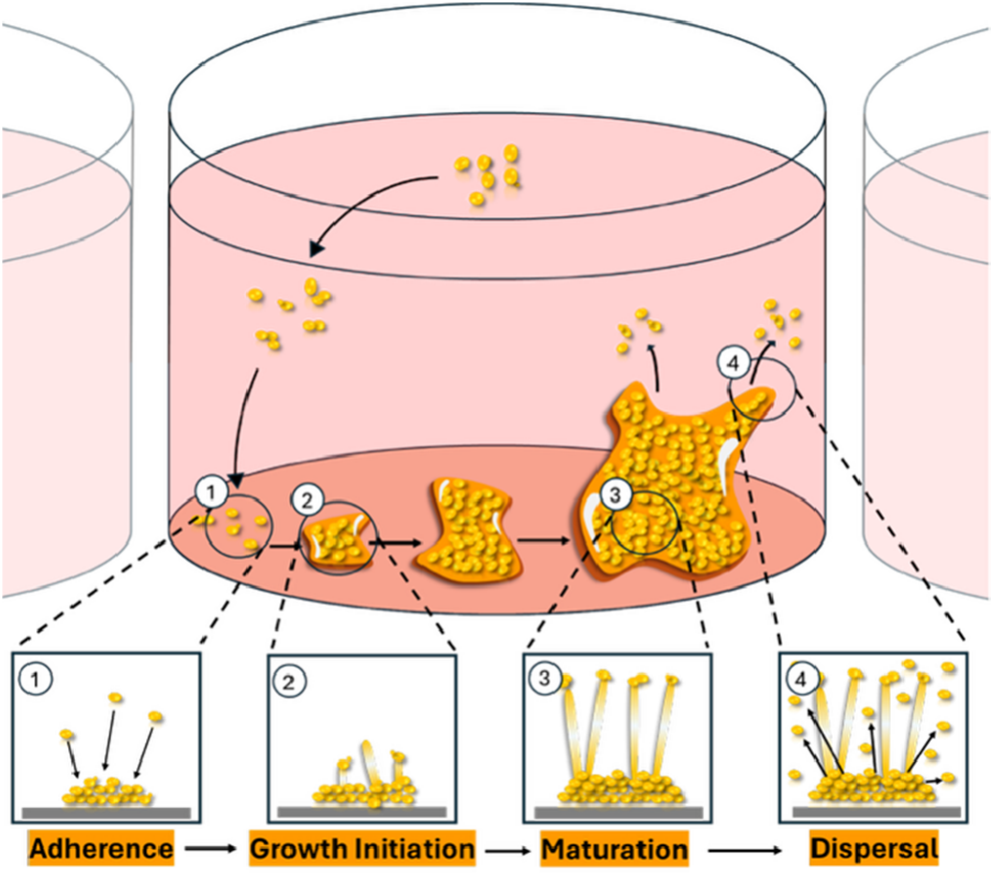
Schematic overview of the biofilm life cycle of *C. albicans*. The biofilm development is illustrated in four consecutive stages: (1) Adherence, (2) growth initiation, (3) maturation, and (4) dispersal.

The ability of *Candida* species to form biofilms enables colonization of mucosal tissues, medical devices such as catheters, as well as other biotic and abiotic surfaces. Thereby, they pose a significant risk to patients, especially in the context of invasive surgeries, promote hospital-acquired transmission, and exhibit increased resistance to antifungal drugs, which together contribute to its high clinical impact.^5–7,15,26,30,39–48^ Therefore, we propose that investigating formation of biofilms and modification in these yeasts is essential for a comprehensive understanding of their pathogenicity and will support the development of improved treatments for *C. albicans*, *C. auris*, and *C. parapsilosis*associated diseases.

There have been numerous studies on the effect of various chemical compounds on biofilm formation.^49,50^

Quaternary ammonium compounds (QACs) are well-established antimicrobial agents: For bacterial envelopes, it has been shown that they exert broad-spectrum activity through electrostatic interactions with the negatively charged cell surface, displacing stabilizing cations such as Ca^2+^ and Mg^2+^.^51^ The interaction is enhanced by interdigitation of the long hydrophobic alkyl chain between the lipids from the double-layer membrane, lowering the fluidity of the membrane. This typically results in membrane disruption, leakage of cellular content, and rapid bactericidal action.^52–56^ Prominent representatives of QACs are the benzalkonium salts—mostly chlorides (BAC)—with two methyl, a benzyl, and a longer alkyl group with 8–18 carbon atoms as substituents (Scheme 1).

**Scheme 1:**
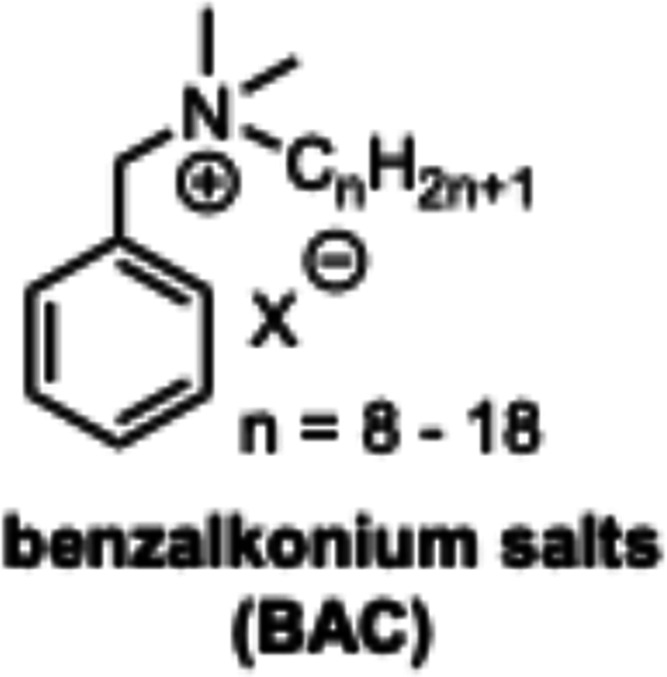
Benzalkonium Salt (BAC) as the Lead Structure for Related Polymeric Quaternary Ammonium Salts (polyQAC) from the Present Study (See Figure 2)

Unfortunately, some bacterial strains have been found to exhibit stable resistance to higher concentrations of small-molecule QACs such as BAC, which in turn could lead to an increased survival rate during disinfection.^57^ In polymeric or surface-immobilized forms, QACs can provide long-lasting coatings that are especially valuable for preventing biofilm formation on materials used in biomedical or environmental settings.^58^ Polymeric QACs (polyQACs) are often studied in microbial experiments to assess not only their direct antimicrobial efficacy but also their effects on biofilm inhibition, microbial adhesion, and resistance development.^59,60^ Their tunable structure (e.g., alkyl chain length, density of quaternary centers) compared to molecular variants allows detailed mechanistic studies and the rational design of advanced antimicrobial materials.^61^ In the literature, structure–activity relationship (SAR) studies on biofilm-inhibiting polymeric quaternary ammonium salts primarily focus on factors like the molecular weight, counterion, spacer length, type of alkyl chain, or polymer architecture.^62,63^ However, the role of local charge distribution and its concomitant influence on hydrophobicity in governing antimicrobial activity and biofilm formation especially of *Candida* species remains insufficiently understood. While QACs have been extensively studied in the context of antibacterial activity, there is a growing but still comparatively limited body of literature addressing their effects on yeasts. Early studies with *Saccharomyces cerevisiae* already suggested that benzalkonium chloride affects yeast cells primarily through membrane-associated mechanisms leading to antifungal activity.^64^ This is consistent with more recent work showing that sterol composition modulates the interaction of benzalkonium chloride and related cationic compounds with yeast membranes, highlighting that QAC yeast interactions are strongly influenced by fungal membrane properties as their sterol content.^65^ In pathogenic yeasts, several gemini QACs and related cationic surfactants have been shown to inhibit adhesion, filamentation, and biofilm formation of *C. albicans* and to exert fungicidal or biofilm-eradicating activity against *Candida* and other yeast-like fungi.^66–68^ However, recent findings on *C. auris* indicate that susceptibility to QAC-containing disinfectants can vary substantially between clinical isolates, emphasizing the need for species-specific investigations.^69^ These studies provide a rationale for examining how structurally defined polymeric QACs interact with clinically relevant biofilm-forming yeasts such as *C. albicans*, *C. auris*, and *C. parapsilosis*. Here, it is important to note that fungal cells are surrounded by a polysaccharide-rich cell wall composed mainly of glucans, chitin, and glycoproteins. QACs are expected to interact first with the cell wall before they may subsequently reach or perturb the plasma membrane.^70,71^ In line with this, studies with cationic surfactants showed high-affinity adsorption to *C. albicans* cells and identified reversal of the cell surface charge, rather than immediate cell lysis, as a critical event in their antifungal activity.^70^

Here, we report three structurally closely related polyQACs synthesized via polymer-analogous reactions^72–76^ between poly­(vinylbenzyl chloride) and the tertiary amines trimethylamine, 1-azaadamantane, or 1-azaadamantan-4-one (Figure 2). These compounds can be regarded as polymeric equivalents of BACs with systematically varied charge distributions and hydrophilicity. Their effects on cell viability and biofilm formation are investigated using *C. auris*, *C. parapsilosis*, and *C. albicans*.

**Figure 2:**
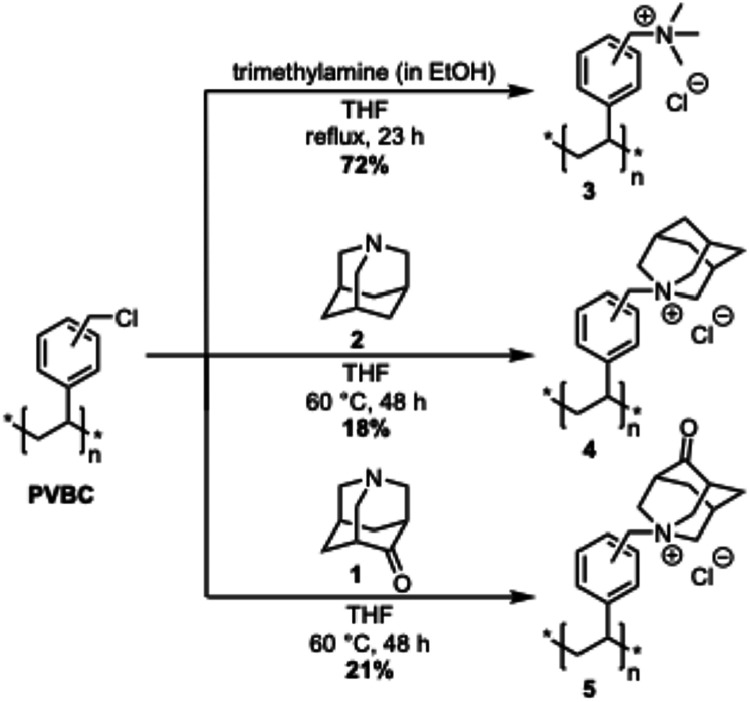
Synthesis of polymers **3**, **4**, and **5** on the basis of PVBC with azaadamantan-4-one (**1**), azaadamantane (**2**), and trimethylamine.

## Experimental Section

### Materials and Methods

Poly­(vinylbenzyl chloride) (PVBC) (*M*_w_ = 100,000 g mol^–1^), trimethylamine in ethanol (4.2 M), resazurin sodium salt, and poly­(vinyl alcohol) (98–99% hydrolyzed and average *M*_w_ 31,000–50,000 g mol^–1^ (PVA40)) have been purchased from Sigma-Aldrich. Poly­(vinyl alcohol) (98% hydrolyzed and average *M*_w_ 16,000 g mol^–1^ (PVA16)), acetic acid, crystal violet, 3-(*N*-morpholino) propanesulfonic acid (MOPS), and paraformaldehyde were acquired from Carl Roth GmbH (Karlsruhe, Germany). RPMI-1640 medium containing l-glutamine was obtained from Thermo Fisher Scientific (Waltham, MA, USA). All other reagents were purchased from ABCR, ACROS-ORGANICS, CHEMPUR, SIGMA-Aldrich, or TCI and used directly without further purification unless otherwise noted. Moisture- or oxygen-sensitive reactions have been carried out in dried glassware, heated under vacuum (10^–3^ mbar), using standard Schlenk techniques in a dry argon atmosphere (Argon 4.6 from MTI INDUSTRIEGASE AG). Anhydrous solvent THF has been obtained from an M. BRAUN solvent purification system (MB-SPS-800) and stored over molecular sieves (3 Å). Other solvents have been purchased and used in analytical or HPLC grade. 1-Azaadamantan-4-one (**1**) has been prepared in accordance to previous synthetic protocols.^77,78^

NMR spectra have been recorded at 298 K, unless otherwise noted, on the following spectrometers: BRUKER *Avance Neo 400* [400.1 MHz (^1^H), 100.6 MHz (^13^C)], BRUKER *Avance III HD 500* [500.3 MHz (^1^H), 125.8 MHz (^13^C)], and BRUKER *Avance Neo 600* with a *Prodigy* CryoProbe [600.2 MHz (^1^H), 150.9 MHz (^13^C)]. ^1^H NMR spectra are referenced to tetramethylsilane as an internal standard or the residual proton signal of the respective solvent: CDCl_3_: δ = 7.26 ppm, D_2_O: δ = 4.79 ppm. ^13^C NMR spectra are referenced to CDCl_3_: δ = 77.16 ppm. Analysis follows first order and the following abbreviations for multiplets are used: singlet (s), doublet (d), multiplet (m), and combinations thereof, *i*.*e*., doublet of doublets (dd). Coupling constants (*J*) are given in Hertz [Hz].

High-resolution mass spectrometry (HRMS) has been performed using a Fourier Transform Ion Cyclotron Resonance (FT-ICR) mass spectrometer solariX (BRUKER DALTONIK GmbH, Bremen, Germany) equipped with a 7.0 T superconducting magnet and interfaced to an Apollo II Dual ESI/MALDI source, which can be switched from ESI to APCI or MALDI operation almost instantaneously. In MALDI operation mode, α-cyano-4-hydroxycinnamic acid (ACCA) has been used as the matrix.

TGA measurements have been carried out with PerkinElmer TGA 8000 with a heating rate of 10 °C min^–1^ under nitrogen flow (20.0 mL min^–1^) or oxygen flow (20.0 mL min^–1^) from room temperature to 800 °C.

DSC measurements have been obtained from a Mettler-Toledo DSC 2 STARe system with a heating rate of 10 °C min^–1^ for three scanning cycles of heating–cooling (in N_2_ and O_2_ atmosphere).

Elemental analyses were performed on an Elementar vario Micro cube.

The water contact angles were measured using the G10 contact angle measurement system (Krüss GmbH, Germany) on a drop-cast film prepared from the polymer solutions (1 mg mL^–1^).

The zeta potential of the polymer solutions was determined using a Nano-Zetasizer (Malvern Instruments) at 25 °C in zeta potential mode. 1 mg of the polymer was dissolved in 1 mL of KCl solution (1 mM). The zeta potentials are reported as the average of three measurements. Using the same solutions and the same apparatus, DLS measurements were performed at 25 °C with a scattering angle of 173° and a wavelength of λ = 633 nm in a polystyrene cuvette.

### Syntheses

For the synthesis of the polymers, the molecular precursors trimethylamine, azaadamantan-4-one (**1**), and azaadamantane (**2**) have been reacted with poly­(vinylbenzyl chloride) (Figure 2).

#### Azaadamantane (**2**)


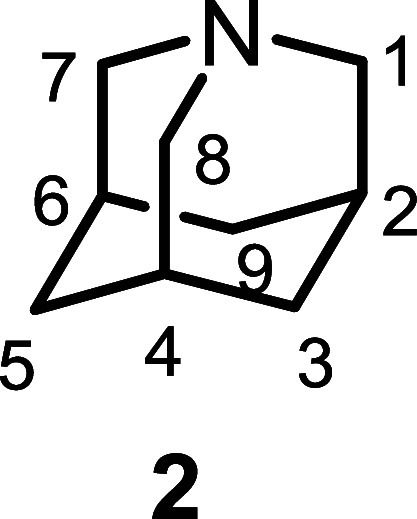
1-Azaadamantan-4-one **1** (1.0 g, 6.6 mmol) and hydrazine hydrate (497.0 mg, 9.9 mmol) are dissolved in TEG (20 mL). Subsequently, potassium hydroxide (1.5 g, 26.5 mmol) is added. The reaction mixture is heated to 200 °C over a period of 15 min followed by stirring at 200 °C for 4 h. The resublimation of the raw product can be observed on the reflux condenser at a temperature of 160 °C. After the reaction is completed, the raw product is washed out of the reflux condenser with diethyl ether. After the evaporation of the solvent under vacuum, the residue is transferred into a separatory funnel by adding 30 mL of 15% sodium hydroxide solution followed by extraction 3 times with 25 mL of diethyl ether. The combined organic phases are washed with 25 mL of 15% sodium hydroxide solution and dried with magnesium sulfate. The solvent is evaporated under vacuum. The obtained residue is purified with a “Kugelrohr apparatus” (120–150 °C, 1–2 mbar) to obtain product **2** (colorless solid, 40% yield, 367.0 mg).

^1^H NMR (400 MHz, CDCl_3_): δ = 3.12 (d, *J* = 1.9 Hz, 1,7,8, 6H), 1.97 (d, *J* = 12.3 Hz, 3,5,9, 3H), 1.91 (d, *J* = 12.3 Hz, 3′,5′,9′, 3H), 1.66 (s, br, 2,4,6, 3H) ppm.

^13^C NMR (101 MHz): δ = 59.21 (1,7,8), 37.01 (3,5,9), 27.75 (2,4,6) ppm.

MS (*m*/*z*): calcd for C_9_H_15_N, [M + H]^+^, 137.12; found, 137.25.

The syntheses of the polyQACs have been carried out according to a recently published protocol.^79^ While polymers **4** and **5** are new, polymer **3**, although known from the literature,^75^ was synthesized there by polymerization from the monomer rather than by a polymer-analogous reaction and is therefore also included.

#### Poly­(vinylbenzyltrimethylammonium chloride) (**3**)


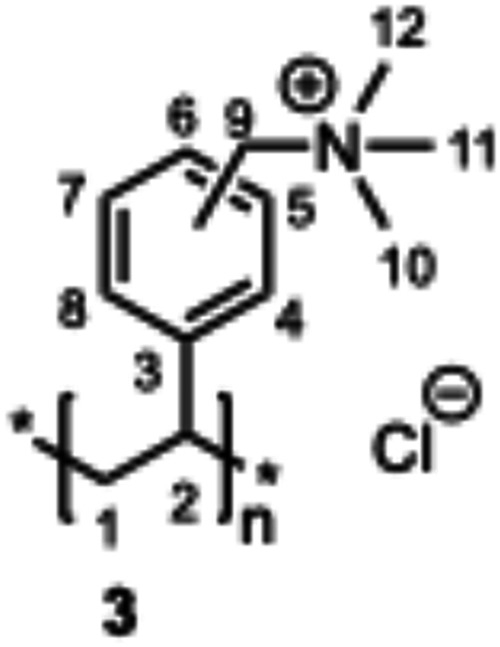
A 50 mL round-bottom flask is charged with poly­(vinylbenzyl chloride) (569 mg, 3.02 mmol, 1.0 equiv), trimethylamine in ethanol (4.2 M) (1.08 mL, 4.54 mmol, 1.5 equiv), and THF (6.0 mL). The reaction mixture is stirred at rt for 23 h. The solvent is removed under reduced pressure and the solid is washed with THF (3 × 10 mL). The residue is dried in *vacuo* to obtain product **3** (450 mg, 2.13 mmol, 72%) as a white solid.

^1^H NMR (400 MHz, D_2_O): δ = 7.13–6.62 (br, 4,5/6,7,8, 4H), 4.33 (br, 9, 2H), 2.95 (br, 10,11,12, 9H), 1.52 (br, 1,2, 3H) ppm.

Solid-state ^13^C NMR (101 MHz): δ = 131.57 (3,4,5,6,7,8), 68.37 (9), 52.59 (10,11,12), 40.23 (2), 25.73 (1) ppm.

#### Poly­(vinylbenzyl-1-azaadamantan-1-ium chloride) (**4**)


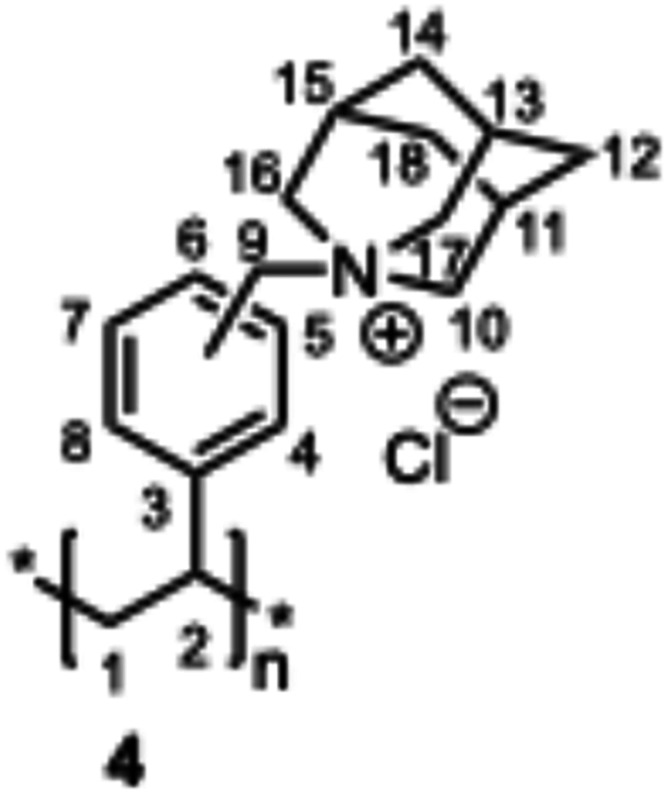
Polymer **4** is synthesized analogously to **3**, from PVBC (80.4 mg, 0.53 mmol) and azaadamantane (**2**) with a slight excess (86.8 mg, 0.63 mmol, 1.2 equiv). For purification, the crude product is dissolved in 10 mL of water and added dropwise to 100 mL of 1,4-dioxane. The resulting white precipitate is isolated by centrifugation at 4000 rpm for 20 min, and the residue is dried *in vacuo* to afford **4** as a colorless solid (27.5 mg, 0.095 mmol, 18% yield).

^1^H NMR (400 MHz, D_2_O): δ = 7.18–6.61 (br, 4,5/6,7,8, 4H), 4.24 (br, 9, 2H), 3.35 (br, 10,16,17, 6H), 2.21–1.77 (br, 1,2,11,12,13,14,15,18, 12H) ppm.

#### Poly­(vinylbenzyl-4-oxo-1-azaadamantan-1-ium chloride) (**5**)


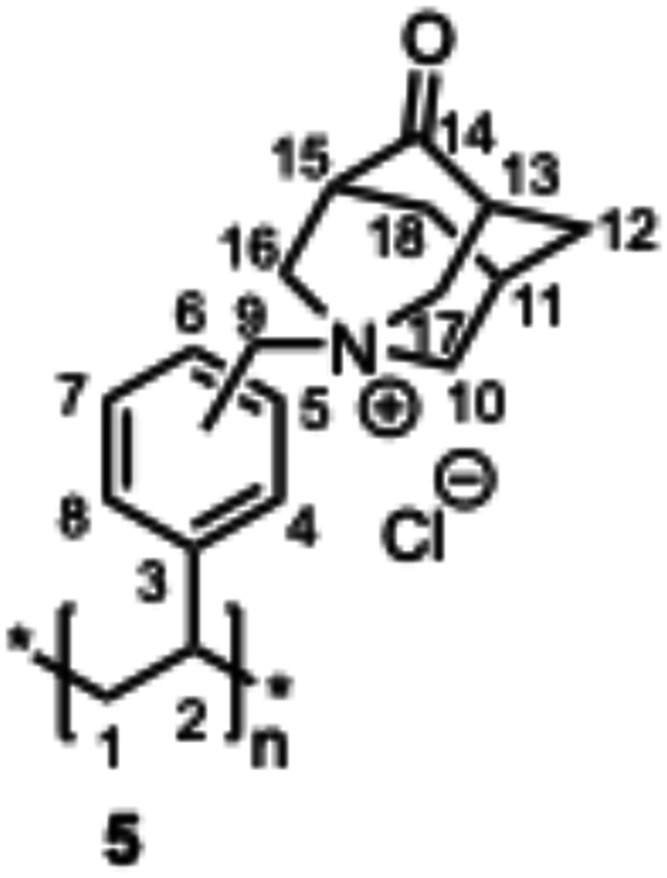
Poly­(vinylbenzyl chloride) (153.0 mg, 1.0 mmol) is dissolved in anhydrous tetrahydrofuran (15 mL) by stirring at 60 °C. After the complete dissolution, azaadamantan-4-one (**1**) (151.0 mg, 1.0 mmol) is added and the reaction mixture stirred for 48 h at 60 °C until a solid precipitate forms. The solvent is evaporated under vacuum. For the purification, the raw product is dissolved in 10 mL of water and added dropwise to 100 mL of tetrahydrofuran followed by centrifugation at 4000 rpm for 20 min. The solution is decanted and the solid dried under vacuum. The purified product **5** is obtained as a colorless solid (63.8 mg, 21% yield).

^1^H NMR (400 MHz, D_2_O): δ = 7.15–6.62 (br, 4,5/6,7,8, 4H), 4.27 (br, 9, 2H), 3.57–3.35 (br, 10,16,17, 6H), 2.20 (br, 2,11,13,15, 4H), 1.71 (br, 1,12,18, 6H) ppm.

### Cell Studies

#### Candida Cultivation

For each experiment, 100 μL of acryoprotectant glycerol culture *C. auris* (DSMZ-No. 21092), *C. parapsilosis* (ATCC22019), or *C. albicans* (ATCC90028) was added in 5 mL of RPMI-1640 medium and incubated at 37 °C for 18 h and orbital-shaking at 150 rpm.

#### Resazurin Reduction Assay/Viability Assay

The viability of the pathogenic yeast cells in the presence of polymers **3**, **4**, and **5** (0–50 μg/mL) and amphotericin B (2 μg/mL) was determined in a manner similar to that described in the guidelines of the Clinical and Laboratory Standards Institute for the M27-A3 broth microdilution test,^80^ by incubating 2.5 × 10^3^ yeast cells in triplicate in 200 μL of compound-enriched RPMI-1640 medium in 96-well flat-bottom polystyrene microtiter plates (Sarstedt AG & Co. KG, Nümbrecht, Germany) at 37 °C for 24 h and under orbital-shaking at 900 rpm on an Eppendorf shaker. Viability was assessed using the resazurin reduction assay.^81,82^ Briefly, cells were incubated for 2 h at 37 °C with 20 μL of a 0.15 mg/mL resazurin solution. Metabolically active cells reduce resazurin to resorufin and fluorescence was measured at an excitation wavelength of 535 nm and an emission wavelength of 595 nm using a Tecan Infinite F200 microplate reader (Tecan Group Ltd., Männedorf, Switzerland). A sterility control was measured in triplicate and subtracted from all readings as the blank.

#### Biofilm Assay

To investigate the effect of polymers **3**, **4**, and **5** (0–50 μg/mL) and amphotericin B (2 μg/mL) on *Candida* biofilm formation in flat-bottom 96-well polystyrene microtiter plates (Sarstedt AG & Co. KG, Nümbrecht, Germany), 2.5 × 10^3^ cells were inoculated in 200 μL of RPMI-1640 medium and incubated at 37 °C without agitation. Biofilm development was quantified after 24 h using the crystal violet assay originally described by George O’Toole^83^ and widely applied to *Candida* spp.^30,84,85^ Briefly, the planktonic phase was removed and the wells were washed twice with 200 μL of water. Biofilms were stained for 15 min with 200 μL of 0.1% (w/v) crystal violet, washed twice with water, air-dried for 24 h at 25 °C and the retained stain was solubilized in 200 μL of 30% acetic acid for 15 min, then transferred into a fresh 96-well plate. Absorbance at 560 nm was measured using a Tecan Infinite F200 microplate reader (Tecan Group Ltd., Männedorf, Switzerland). A sterility control was measured in triplicate and subtracted from all readings as the blank.

To assess the effects of the polymers and their respective starting materials on *C. auris*, *C. parapsilosis*, and *C. albicans* biofilm formation on glass, the procedure described was used with small modifications. In summary, HPLC glass vials were incubated for 24 h at room temperature with 150 μL of polymer solution (50 μg/mL in water) to allow the compounds to coat the glass surface. As a nontreated reference, water has been applied to the HPLC vials in the same manner. In the next step, the vials were inoculated with 150 μL of RPMI-1640 medium containing 2.5 × 10^3^ *Candida* cells and incubated at 37 °C under static conditions. Subsequently, the vials were washed twice with 150 μL of water. Biofilms were stained for 15 min with 150 μL of 0.1% (w/v) crystal violet, washed twice with water, air-dried for 24 h at 25 °C, and the retained stain was solubilized in 150 μL of 30% acetic acid for 15 min. The resulting mixture was then transferred into a fresh 96-well plate. Absorbance at 560 nm was measured using a Tecan Infinite F200 microplate reader (Tecan Group Ltd., Männedorf, Switzerland). A sterility control was measured in triplicate and subtracted from all readings as the blank.

## Results and Discussion

### Polymer Synthesis and Characterization

To broaden the range of polyQACs studied for their antimicrobial effects, three positively charged polymers (**3**, **4**, and **5**) have been synthesized in accordance to a recently published protocol (Figure 2).^79^ Purification by reprecipitation from THF or 1,4-dioxane has been performed to remove unreacted or only partially functionalized starting material, affording the polymers in yields between 18% and 72%. The moderate yields may be related to the small reaction scale and subsequent relatively higher loss, especially during purification. Molecular weights cannot be reliably determined by GPC—as already reported in the literature for similar compounds,^86^ or HR-MALDI mass spectrometry.

Instead, the polymer structures are confirmed by ^1^H NMR spectroscopy and are consistent with those of related compounds (Figure 3).^87^ While the aromatic protons at 7.1 and 6.6 ppm remain unaffected by the functionalization with tertiary amines, the benzylic methylene groups show a high-field shift from 4.6 to 4.3 ppm. The most significant change is evident in the appearance of new peaks between 3.6 and 3.0 ppm, which can be attributed to the (CH_2_)_3_N^+^ or (CH_3_)_3_N^+^ protons. The peaks between 2.2 and 1.5 ppm correspond to the remaining cage and backbone protons. A more detailed assignment can be found in the experimental section. Integration of the new peaks allows for determination of the molar degree of functionalization (DoF). Because of the overlap of the benzylic resonances of the functionalized and unfunctionalized groups, determining the DoF based on this signal would be highly inaccurate. Therefore, the DoF was instead calculated from the integral ratio of the aromatic protons to that of the (CH_2_)_3_N^+^ or (CH_3_)_3_N^+^ protons. The spectra were integrated over the entire range from 2.3 to 3.7 ppm for polymer **3** and from 2.7 to 3.9 ppm for polymers **4** and **5**. This analysis indicates an essentially quantitative functionalization, with DoF values between 91% and 98% (Table 1), consistent with the literature values for related polymers.^79^

**Figure 3:**
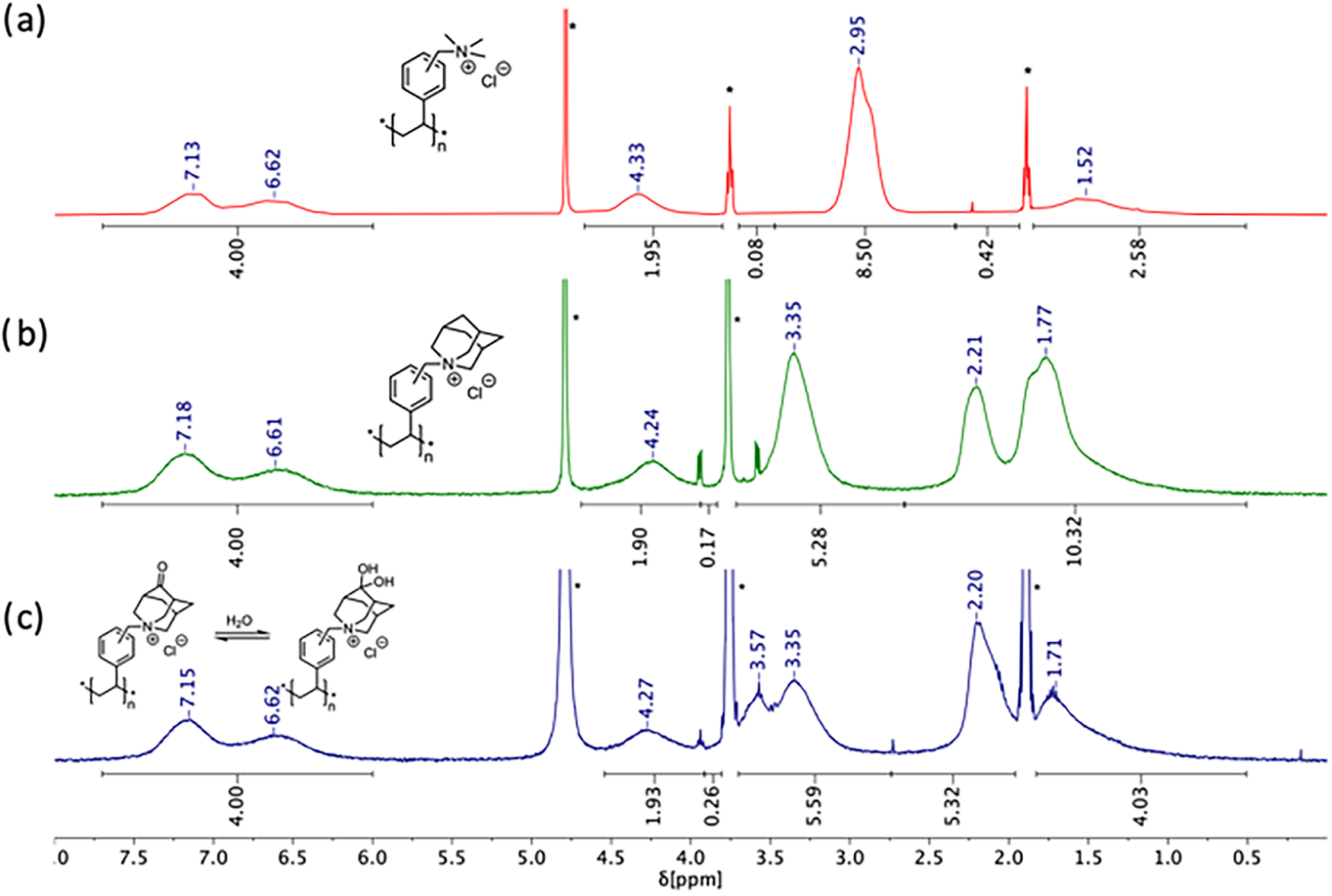
^1^H NMR spectra of polymers (a) **3**, (b) **4**, and (c) **5** in D_2_O obtained by polymer-analogous reactions. *Residual solvent THF and dioxane and NMR solvent (HDO).

**Table 1:** Elemental Analysis of the Polymers **3**, **4**, and **5** Obtained by Polymer-Analogous Reactions

polymer	formula^a^	calculated (%)	found (%)	DoF (%)
**3**	(C_12_H_18_NCl)_400_(H_2_O)_600_	C 60.44, H 9.06, N 5.37	C 60.44, H 9.21, N 5.43	100
**4**	(C_18_H_24_ClN)_1632_(C_9_H_15_N)_787_	C 61.47, H 9.25, N 4.44	C 61.47, H 9.25, N 4.44	100
	(C_4_H_8_O_2_)_649_(H_2_O)_6933_			
**5**	(C_18_H_22_ClNO)_3012_(C_9_H_9_Cl)_174_	C 63.29, H 7.76, N 3.91	C 63.29, H 7.76, N 3.91	95
	(C_4_H_8_O)_268_(H_2_O)_6546_			

a C_18_H_22_NClO, C_18_H_24_NCl, or C_12_H_18_NCl: functionalized PVBC; C_9_H_15_N: residual excess of azaadamantane; C_4_H_8_O, C_4_H_8_O_2_, or H_2_O: residual solvent (THF, dioxane, or water) from workup.

To further substantiate the results of the NMR analysis, the DoF was also determined by elemental analysis. It should be noted that the polymers are highly hygroscopic due to the quaternary ammonium groups, which promote strong interactions with the polar solvents used during workup. In addition, since polymer **5** readily forms a hydrate in the presence of moisture (see above),^88^ even prolonged drying under vacuum at an elevated temperature does not ensure complete removal of residual THF, 1,4-dioxane, or water. Considering these solvent contributions (see the Supporting Information), elemental analysis yields DoF values between 95% and 100%, confirming the essentially quantitative functionalization (Table 1).

Additionally, DoF has been estimated from thermogravimetric analysis (TGA) (see the Supporting Information). The thermograms reveal a mass loss up to 200 °C, attributed to the evaporation of residual solvents (see above). A pronounced mass decrease between 200 and 600 °C is observed, corresponding to the decomposition of the quaternary ammonium salts and partial degradation of the parent PVBC. The latter shows mass loss of 65% up to 600 °C.^89^ Comparison with the experimental mass loss of polymers **3**, **4**, and **5** delivers essentially quantitative DoF values (see the Supporting Information and Table 2).

**Table 2:** Comparison of the Molar Degree of Functionalization (DoF, in %) Determined by ^1^H NMR Spectroscopy, Elemental Analysis (EA), and Thermogravimetry (TGA)

polymer	NMR	EA	TGA
**3**	95	100	100
**4**	91	100	100
**5**	98	95	100

All three methods indicate an almost quantitative functionalization, confirming the well-defined structure of these materials. The glass transition temperatures (*T*_g_) of polymers **3** and **4**, observed at 101 and 104 °C, respectively, are comparable to that of pure polystyrene. For polymer **5**, no reliable *T*_g_ can be determined due to its pronounced hygroscopicity (see above and the Supporting Information).

To investigate the hydrophilic properties of the polymers, the time-dependent static water contact angles of drop-cast films were measured (Figure 4). Since all polymers are water-soluble, the initially high contact angle of 70–80° decreases rapidly. The curves can be satisfactorily modeled by a double exponential decay, suggesting at least two phases: an initial rapid phase with a decay constant *k*_1_ between 0.14 and 0.23 s^–1^, followed by a significantly slower second phase with *k*_2_ between 0.016 and 0.030 s^–1^. We assume that the first step is caused by wetting-induced swelling of the polymer while the second is due to the dissolution of the polymer molecules. The contact angles start between 70 and 82° and reach final values of 9–23° (Table 3). While the initial values do not differ significantly, the final angles show a clear trend indicating a defined structure–property relationship with respect to hydrophilicity (see discussion below). In addition, the zeta potential was determined, which, as expected, exhibited an overall positive value but showed only minor differences between the three polymers. It ranges from 52 to 58 mV (Table 3).

**Figure 4:**
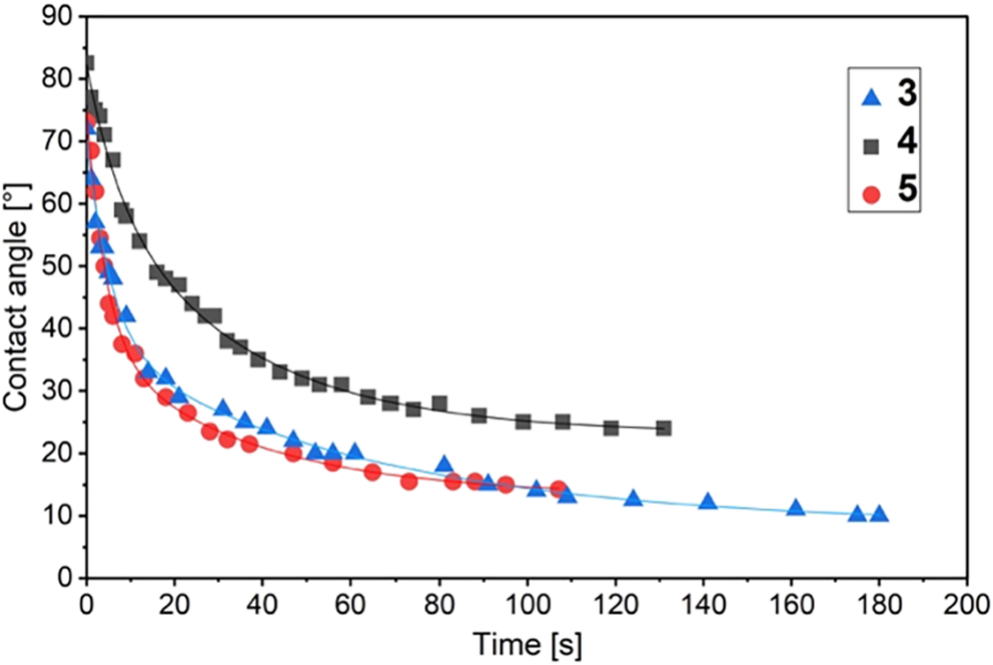
Water contact angles of the polymers on drop-cast films.

**Table 3:** Kinetic Parameters of Time-Dependent Contact Angle and Zeta Potential (See Figure 4)

polymer	ζ/mV	k_1_/s^–1^	k_2_/s^–1^	start angle	final angle
**3**	52.3 ± 3.2	0.18 ± 0.02	0.016 ± 0.003	70 ± 5	8.6 ± 1.3
**4**	53.7 ± 3.0	0.14 ± 0.04	0.030 ± 0.005	82 ± 12	23.2 ± 1.0
**5**	58.3 ± 3.0	0.23 ± 0.03	0.029 ± 0.003	75 ± 8	13.2 ± 1.5

The polymer structures were inspired by BAC as a template (see Scheme 1), with polymer **3** serving as the starting point. Although BACs typically contain at least one C_8_ or longer alkyl substituent, only methyl groups were introduced, as the longer hydrophobic chains, in combination with the hydrophobic polystyrene backbone, could reduce solubility in aqueous environments too significantly. The small methyl groups can rotate freely around the C–N bond and keep the positive charge readily accessible to countercharges. In contrast, the 1-azaadamantane substituent in polymer **4** is larger and capable of distributing the positive charge within the rigid carbon cage via hyperconjugation through σ bonds. This shields the charge significantly and makes the substituent more hydrophobic than the trimethyl groups in **3**. The introduction of a keto group into the carbon cage in polymer **5** counteracts the shielding effect and makes it more hydrophilic through dipole–dipole interactions, as it quantitatively forms the hydrate in water (see Figure 3a inset).^88^ This results in geminal hydroxyl groups that can form strong hydrogen bonds with suitable acceptors. These structural considerations are confirmed by the resulting contact angles, with the smallest value measured for the most hydrophilic polymer **3**, followed by the highest value for the least hydrophilic compound **4** and an intermediate value for polymer **5** (see Table 2). The shielding effect of the molecular cages on the charge is only slight. It should be noted that the polymers tend to form aggregates at the concentrations used, as recognized by DLS measurements (not shown); these aggregates are presumably strongly influenced by the cell medium, making the measured potential values even less meaningful.

Another reason for using azaadamantanes as substituents is the fact that nitrogen-containing derivatives of adamantane are extremely attractive amines that serve as promising scaffolds for medicinal chemistry and exhibit proven antimicrobial activity.^90–95^ The structural similarity of these polymers to BAC, along with their thermal stability up to 200 °C and their water solubility of over 15 mg/mL, make them promising candidates for biological applications.

### Cell Experiments

Polymeric compounds can exert diverse effects on microorganisms ranging from the induction of phenotypes typically expressed only within multicellular clusters of bacteria to antibiofilm activity in *Candida* species.^96,97^ To assess the effect of polymer **3** on *C. albicans*, *C. auris*, and *C. parapsilosis*, planktonic viability and biofilm formation were investigated separately. In the initial set of experiments, polymer **3** was administered in solution under conditions preventing biofilm formation, allowing to independently evaluate its effect on planktonic cells. No significant effect of polymer **3** on cell viability is observed for the tested *Candida* species in comparison to the untreated control. In contrast, Amphotericin B at a concentration of 2 μg/mL completely reduces cell viability and serves as a positive control (Figure 5).

**Figure 5:**
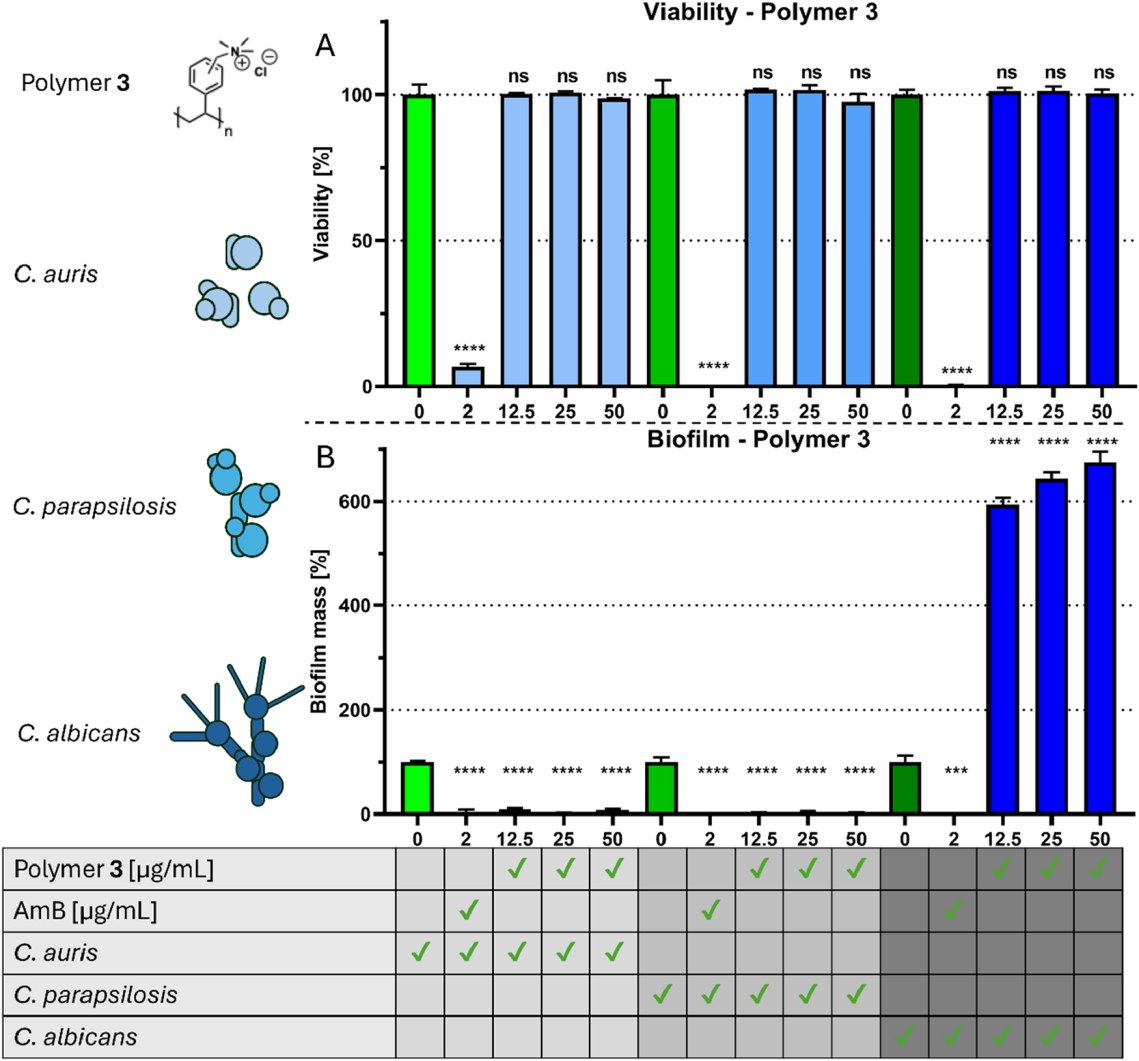
(A) Viabilities of *C. auris*, *C. parapsilosis*, and *C. albicans* cells incubated in 96-well plates with polymer **3** at different concentrations and 2 μg/mL of Amphotericin B as a control. Untreated cells (green) serve as a reference and are set to 100% cell viability. All experiments have been performed in triplicate. (B) Biofilm quantification of *C. auris*, *C. parapsilosis*, and *C. albicans* cells incubated in 96-well plates with polymer **3** at different concentrations and 2 μg/mL of Amphotericin B as a control. Untreated cells (green) serve as a reference and are set to 100% biofilm mass. Statistical significance was assessed by one-way ANOVA followed by Dunnett’s multiple comparisons test versus the untreated reference of each strain. Significance levels are indicated as ***p < 0.001, ****p < 0.0001; n.s., not significant.

As the ability to form robust biofilms contributes significantly to the pathogenesis of the three tested pathogenic yeasts,^27,29,30,35,39,45,47,48,98,99^ we have performed a subsequent series of experiments to evaluate the impact of the polymer on their biofilm formation. Therefore, a constant density has been seeded in a microtiter plate and incubated with polymer **3** for 24 h. In contrast to the viability assay, the experimental setup allowed biofilm formation. In the presence of polymer **3**, the biofilm formation of both *C. auris* and *C. parapsilosis* could be fully suppressed even when exposed to the lower tested concentration of 12.5 μg/mL. Surprisingly, a pronounced effect to the opposite side is observed when polymer **3** is applied to *C. albicans*: Biofilm formation is increased to 674% compared to the untreated control (Figure 5B).

The finding that an agent can generally impact *Candida* biofilm formation while planktonic cells remain (mostly) unaffected has been reported previously.^81^ Low concentrations of peptide POM1 (and derivatives) reduce *C. albicans* biofilm formation without affecting planktonic cells. Furthermore, there are fundamental differences in the biofilm formation and structure among the three tested pathogens: *C. albicans* is known to build the largest and most complex biofilms among them with a well-defined extracellular matrix in which hyphae and agglutinin-like sequence (Als) proteins have a central role.^38,100,101^ *C. auris* biofilms are known to be thinner and less robust. Additionally, their repertoire of ALS and other adhesin genes is significantly smaller than that of *C. albicans*.^25,100^ *C. parapsilosis* builds biofilms of a similar size to *C. auris*, which in contrast consists mainly of clumped blastospores.^26,38^ In general, they differ in their genetic background and gene expression profiles, leading to differences in the biofilm composition and thickness.

Our findings suggest that polymer **3** fully inhibits *C. auris* and *C. parapsilosis* biofilm formation even at a lower tested concentration of 12.5 μg/mL, while it enhances biofilm formation for *C. albicans* at all tested concentrations. Although interactions of the positively charged polymer **3** with the fungal cell surface are likely involved, the present data do not allow the identification of specific molecular targets explaining the species-specific biofilm inhibition. Instead, the observed species-dependent effects may arise from differences in the physicochemical interactions between the DLS confirmed polymer aggregates and the respective fungal cell surfaces. For cells of *C. albicans*, the polymer likely functions as a surface-associated bridging agent that enhances cell–cell aggregation in the planktonic phase without blocking the specific adhesion proteins. These species-dependent differences once again highlight the fundamental differences between the biofilm composition and properties between the different *Candida* species as described above.

To determine how biofilm formation is influenced by subtle structural variations and changes in the charge distribution and hydrophilicity of the polymers, cell studies were conducted with polymer **4**, in which the methyl groups are replaced by the rigid adamantane cage (see Scheme 1 and Figure 2). The same experimental setup was used as for polymer **3**, with the growth of planktonic cells and biofilm formation being analyzed separately. In line with the results obtained for polymer **3**, no significant effect of polymer **4** on planktonic cells of *C. auris* and *C. parapsilosis* was detected. Although a statistically significant reduction in viability was observed at 50 μg/mL for *C. albicans*, the absolute decrease was minor, with cell viability remaining at 96%. This suggests that the effect, while statistically detectable, is unlikely to represent biologically relevant cytotoxicity under the tested conditions, indicating that polymer **4** likewise does not exhibit activity against planktonic growth in this species (Figure 6). Although the adamantane cage in **4** can be considered more hydrophobic than the methyl groups in **3**, its incorporation into the cell membrane is presumably suppressed for steric reasons.

**Figure 6:**
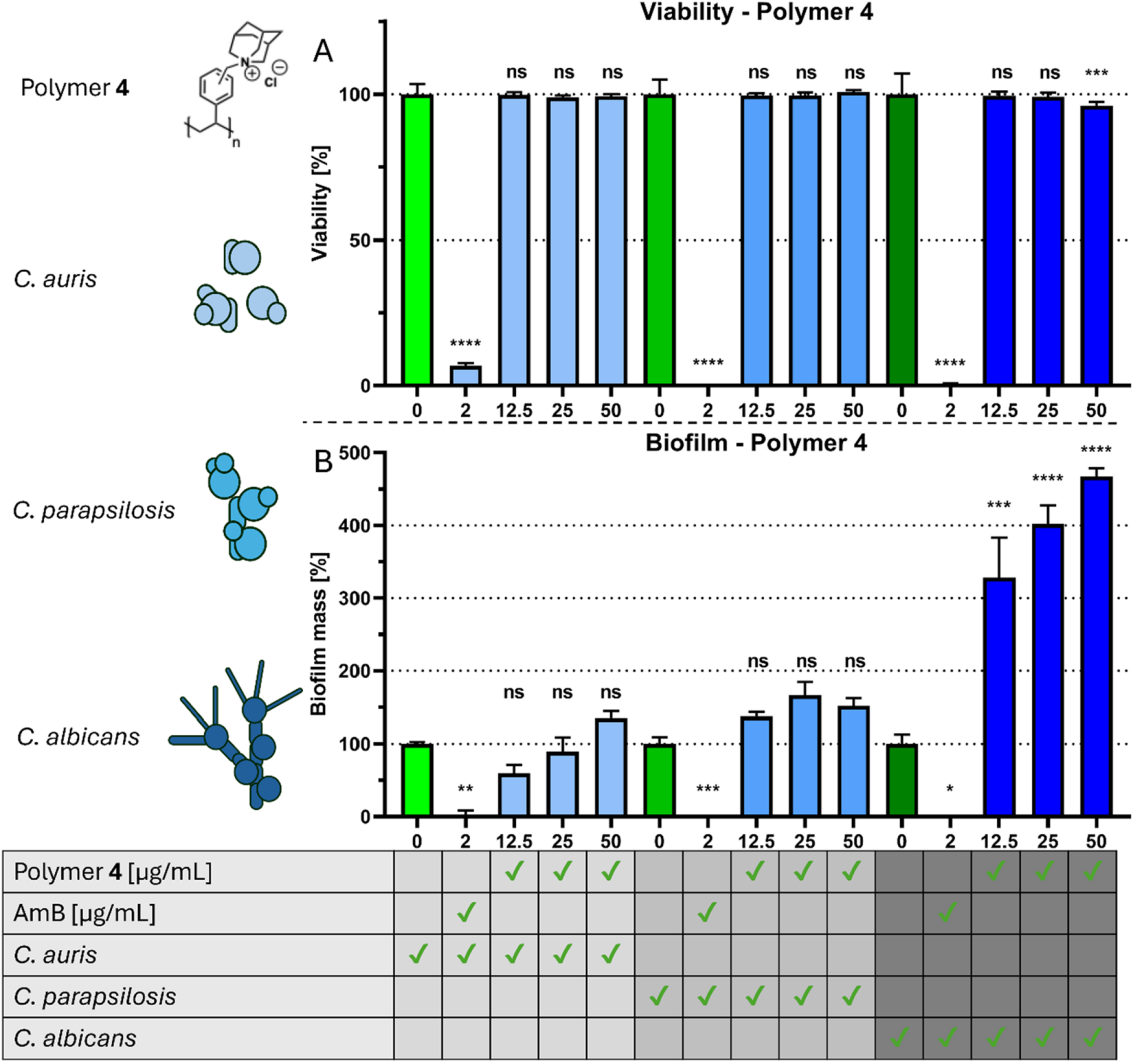
(A) Viability of *C. auris*, *C. parapsilosis*, and *C. albicans* cells incubated in 96-well plates with polymer **4** at different concentrations and 2 μg/mL of Amphotericin B as a control. Untreated cells (green) serve as a reference and are set to 100% cell viability. (B) Biofilm quantification of *C. auris*, *C. parapsilosis*, and *C. albicans* cells incubated in 96-well plates with polymer **4** at different concentrations and 2 μg/mL of Amphotericin B as a control. Untreated cells (green) serve as a reference and are set to 100% biofilm mass. Statistical significance was assessed by one-way ANOVA followed by Dunnett’s multiple comparisons test versus the untreated reference. Significance levels are indicated as *p < 0.05, **p < 0.01, ***p < 0.001, ****p < 0.0001; n.s., not significant.

In contrast to polymer **3**, polymer **4** did not significantly modulate the biofilm formation of *C. auris* nor *C. parapsilosis*. For *C. albicans*, biofilm formation increased significantly with rising polymer **4** concentration, reaching 467% of the control (Figure 6). Since **4** is less hydrophilic than **3**, because of the shielded charge, it likely binds less strongly to the cell surface and therefore has less pronounced effects. The concentration-dependent effects observed for *C. albicans* may reflect changes in polymer–cell and cell–cell interactions resulting from the physicochemical properties as cooperativity of polymer **4**. Such interactions could promote cellular aggregation and thereby indirectly influence the subsequent *C. albicans* biofilm development.

To compensate for the increased hydrophobicity of the adamantane cage in **4** compared to **3**, a carbonyl group was introduced in polymer **5**, resulting in a hydrophilic moiety with two hydroxy groups due to the presence as hydrate in water (see Figure 3c).

Polymer **5** was evaluated using the same experimental procedure as that for polymers **3** and **4**. Similar to polymers **3** and **4**, planktonic growth of *C. auris*, *C. parapsilosis*, and *C. albicans* remained unaffected by its presence (Figure 7).

**Figure 7:**
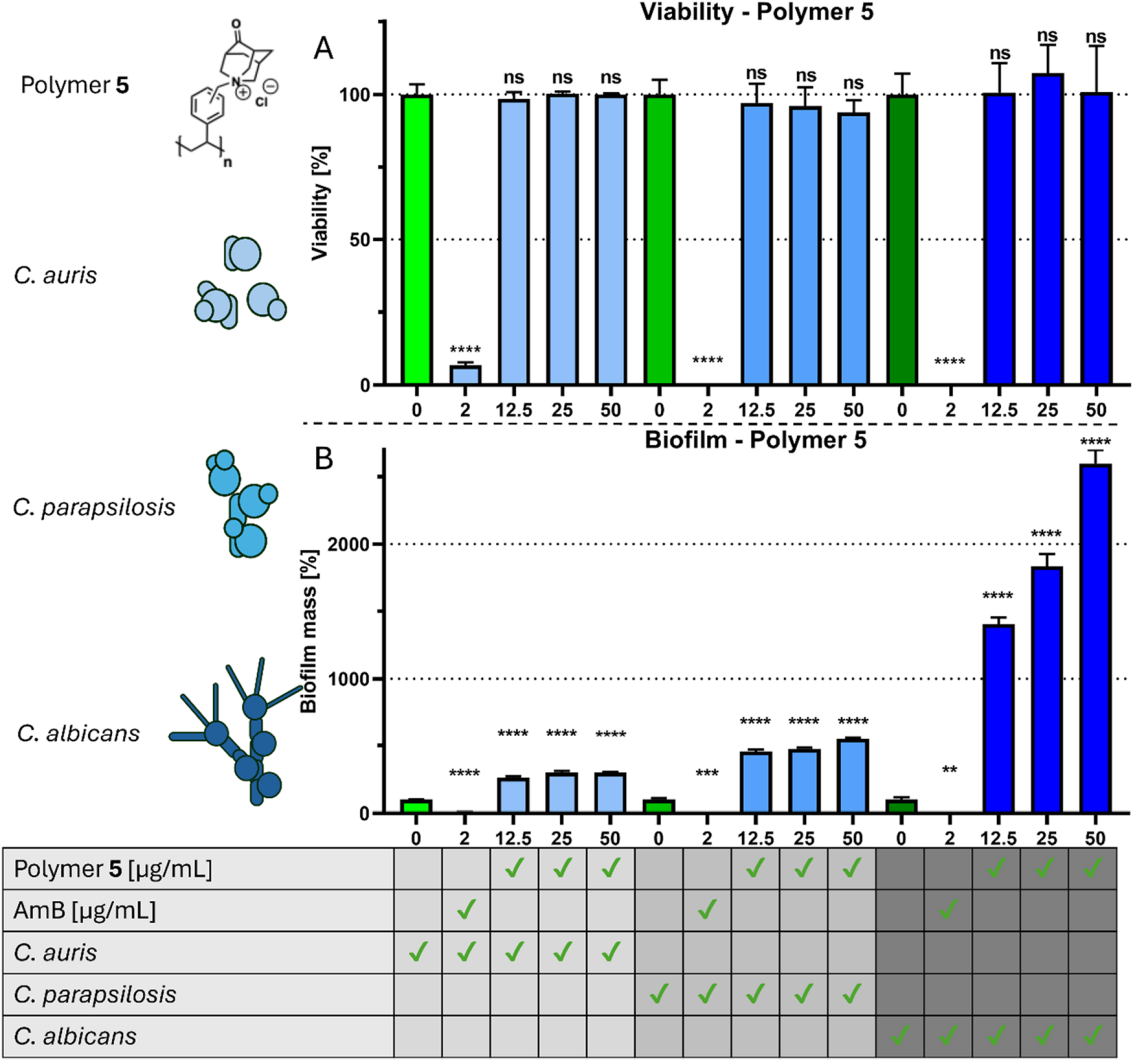
(A) Viability of *C. auris*, *C. parapsilosis*, and *C. albicans* cells incubated in 96-well plates with polymer **5** at different concentrations and 2 μg/mL of Amphotericin B as a control. Untreated cells (green) serve as a reference and are set to 100% cell viability. (B) Biofilm quantification of *C. auris*, *C. parapsilosis*, and *C. albicans* cells incubated in 96-well plates with polymer **5** at different concentrations and 2 μg/mL of Amphotericin B as a control. Untreated cells (green) serve as a reference and are set to 100% biofilm mass. Statistical significance was assessed by one-way ANOVA followed by Dunnett’s multiple comparisons test versus the untreated reference. Significance levels are indicated as **p < 0.01, ***p < 0.001, and ****p < 0.0001; n.s., not significant.

In contrast, polymer **5** had a pronounced effect on biofilm formation of the pathogens. Biofilm biomass reached 300% for *C. auris* and 550% for *C. parapsilosis*. Furthermore, **5** further surpasses the already strong effects of **3** and **4** on *C. albicans* biofilm formation, resulting in biofilm enhancement of 1406% at 12.5 μg/mL, 1835% at 25 μg/mL, and 2598% at 50 μg/mL (Figure 7). Presumably, the hydroxy groups promote stronger binding to the cell wall via the formation of hydrogen bonds and thus aggregation in the planktonic phase, which could exceed any adhesion reduction or blocking and hence accelerates adhesion to the polystyrene surface, facilitating rapid biofilm formation.

To evaluate whether the biofilm enhancing or inhibitory effects of the polymers primarily occur when they are present in solution, a modified experimental design with preadsorption of the polymers to the substrate walls was employed. In this case, glass vials were chosen because of the negatively charged surface (≡Si–O^–^ groups). HPLC glass vials have been incubated with solutions of the respective polymer compounds for 24 h to allow adsorption onto the glass surface. In the second phase, the vials were incubated with cells and biofilm formation was quantified after further 24 h of incubation. Since the cells are incubated in the absence of dissolved polymers in the medium, these experiments specifically address the adherence phase of the planktonic cells on glass and less the aggregation of planktonic cells with each other. Moreover, glass is a surface that is inherently different from polystyrene and might be more difficult to be addressed by colonization.^103,104^ Biofilm formation by *C. auris* and *C. parapsilosis* on glass was not significantly affected by polymers **3**–**5**. For *C. albicans*, polymers **3** and **4** had significant biofilm-enhancing effects of 30% and 74%, respectively. Polymer **5** exhibits once again the strongest effect on *C. albicans* enhancing biofilm formation on glass reaching a level as high as 838% (Figure 8).

**Figure 8:**
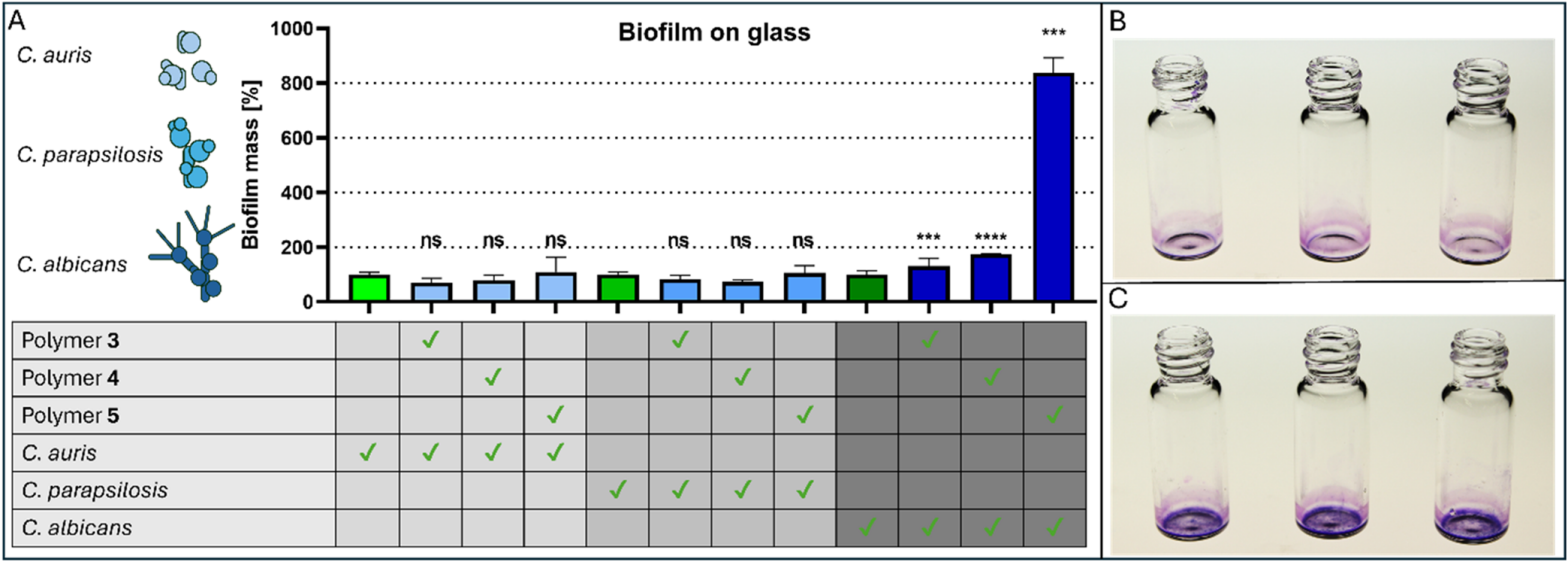
(A) Biofilm quantification of *C. auris*, *C. parapsilosis*, and *C. albicans* cells incubated in HPLC glass vials that have been treated with polymers **3**, **4**, and **5** (blue) at a concentration of 50 μg/mL. Control HPLC glass vials have been treated with plain water and have been set to 100% biofilm mass (green). All experiments have been performed in triplicate. (B) HPLC glass of untreated samples after *C. albicans* biofilms have been stained with crystal violet. (C) HPLC glass of polymer **3** treated sample after *C. albicans* biofilms have been stained with crystal violet. For *C. auris* and *C. parapsilosis*, statistical significance was assessed by one-way ANOVA followed by Dunnett’s multiple comparisons test versus the untreated reference. Significance levels are indicated as n.s., not significant. For *C. albicans*, the shown data was derived from multiple independent data sets, each with its own untreated reference control; therefore, an unpaired *t* test was used to assess significance.

The observed differences between the experiments performed with polymer in solution and on glass surfaces provide important insights into the possibly underlying mechanism of biofilm modulation. While polymer **3** inhibited biofilm formation of *C. auris* and *C. parapsilosis* in solution, these effects were largely absent on glass surfaces, suggesting that the inhibition is not exclusively mediated by direct surface interactions. DLS measurements revealed polymer aggregate formation, indicating that these aggregates may act as physicochemical interaction platforms for fungal cells. *C. auris* and *C. parapsilosis* may therefore preferentially interact with polymer aggregates suspended in solution rather than with the underlying surface, resulting in reduced surface-associated biofilm formation. Consequently, the observed inhibition may arise from aggregate-mediated sequestration or redistribution of cells rather than from specific inhibition of adhesion processes. In contrast, *C. albicans* consistently exhibited enhanced biofilm formation. This species possesses unique biological features, including hyphal morphogenesis and the ability to form highly structured biofilms with complex extracellular matrices, which may enable more efficient surface colonization and promote beneficial cell–cell or cell–polymer interactions. Notably, all three polymers also significantly increased *C. albicans* biofilm formation on glass surfaces, although less strongly than in the microtiter plate experiments. This suggests that the response *of* *C. albicans* can not only be explained by interactions with polymer aggregates in solution and that additional surface-associated mechanisms may contribute. Taken together, the available data suggests that physicochemical interactions between fungal cells and polymer aggregates play a substantial role in determining the observed biofilm phenotypes. However, species-specific biological properties, particularly those of *C. albicans*, are also likely to contribute to the observed responses.

To gain a more fundamental understanding of the pronounced effect of polymer **5** on biofilm formation, the entire experimental setup was repeated with reference compounds that serve as representatives of key structural elements of **5**. These include the azaadamantanone precursor **1** as well as poly­(vinyl alcohol) with two different molecular weights (16,000 (PVA16) and 31,000–50,000 g mol^–1^ (PVA40)). The azaadamantane derivative **1** can be regarded as a characteristic monomer unit of **5** (see Figure 3a, inset), which also has two geminal hydroxy groups. Poly­(vinyl alcohols) are uncharged polymeric analogues with a high density of hydroxyl groups, which are intended to provide information about the effect of hydroxyl groups without existing charges and the influence of molecular weight. As for polymers **3**, **4**, and **5**, the reference compounds exerted no distinct effect on the viabilities of *C. auris*, *C. parapsilosis*, and *C. albicans* (Figure S1). None of the tested compounds clearly reduced biofilm formation of the pathogenic fungi on polystyrene 96-well plates. Instead, a tendency toward biofilm enhancement was noted (Figure S2). On glass surfaces, the effect rather shifted to a decrease in biofilm mass (Figure S3).

All three polymers tested show no significant effect on planktonic *C. auris*, *C. parapsilosis*, and *C. albicans* cells, as the absence of long alkyl chains means that there is no clear amphiphilicity and therefore no possibility of anchoring in the cell surface/membrane. In contrast, polymer **3** completely abolished the biofilm formation of *C. auris* and *C. parapsilosis* cells. Biofilm formation by *C. albicans* was strikingly potentiated most efficiently by polymer **5** and also in a highly significant way for **3** and **4**. A similar but albeit less pronounced potentiation by polymer **5** was observed for *C. auris* and *C. parapsilosis*. On glass surfaces, the effects on biofilm formation were comparably modest for *C. auris* and *C. parapsilosis*, whereas *C. albicans* again displayed strong modulation. The ability to form hyphae, which is unique to *C. albicans* and absent to *C. auris* or *C. parapsilosis*, could be one of the main reasons for the pronounced biofilm enhancement, which could only be observed for *C. albicans* cells. However, this hypothesis requires direct microscopic validation. The moderate effect of the polymers preadsorbed onto the glass is mainly due to the amount of approximately 1 μg per vial (see Figure S4), which was estimated from adsorption experiments with silicon dioxide particles and is 2–20 times lower than in the experiments in solution. As no significant changes in cell viability were detected, the presence of the polymers does not appear to affect the total cell numbers. Moreover, the pronounced differences suggest that the polymers modulate the balance between biofilm formation and persistence in the planktonic phase.

Since the positively charged polymers **3** and **4** and especially polymer **5** show clear effects on biofilm formation, a mechanistic structure–property relation (SPR) for the interaction between the materials and the *Candida* cells is proposed. Thus, the species-dependent response to the polyQACs likely reflects differences in fungal surface architecture rather than differences in planktonic susceptibility, consistent with known species-specific variation in cell-wall architecture among *Candida* species.^105^ Fungal cell walls generally consist of an inner β-glucan/chitin network and an outer mannoprotein-rich layer, but the exposure and relative abundance of these components differ between species.^70^ Since the polyQACs are positively charged, their first interaction is expected to occur at the cell wall rather than directly at the plasma membrane.^71^ In this context, differences in surface charge, mannan composition, β-glucan exposure, and cell-wall protein presentation may affect polymer adsorption.^71,106^ The highly dynamic cell wall of *C. albicans*, particularly during morphogenetic switching, may provide multiple accessible binding sites for polycationic polymers, whereas the distinct surface organization of *C. auris* and *C. parapsilosis* may result in different adsorption patterns.^106–108^ Therefore, the species-dependent effects observed here are likely not only related to biofilm morphology but also to differences in the molecular organization and accessibility of the fungal cell wall. We assume that an interplay of (i) the positive charge, (ii) the capability of hydrogen bonding by the hydroxy groups, and (iii) the cooperativity through the polymeric structure is responsible for the interaction with the cells. Tentatively, the polymers act as anchors at the negatively charged cell surface. The divergent responses observed among the three Candida species indicate that the effects of the polyQACs cannot be explained by a single universal mechanism. Rather, the polymers appear to modulate species-specific steps of biofilm development. In *C. auris* and *C. parapsilosis*, polymer **3** strongly reduced biofilm formation, suggesting that the accessible cationic groups may interfere with initial surface adhesion or mask cell-wall-associated adhesins. This interpretation is consistent with the comparatively thinner and less complex biofilms formed by these species, as well as with their distinct adhesin repertoires and surface architectures. In contrast, *C. albicans* responded to all polymers with enhanced biofilm formation, most prominently to polymer **5**. This species is characterized by a more complex biofilm architecture, including hyphal growth, extensive extracellular matrix formation, and a broad set of adhesins. Therefore, in *C. albicans*, the polymers may act predominantly as multivalent bridging or anchoring agents that promote cell–cell aggregation and surface colonization rather than blocking adhesion. The azaadamantane cage in polymer **4** weakens the ionic interactions and thus binds less efficiently to the proteins with a subtle equilibrium between biofilm inhibition and enhancement. Introducing hydrogen-bonding capabilities strengthens the anchoring to the surface, which promotes aggregation and biofilm formation. The multiple focal points along the polymeric chains increase the effects compared to the monomeric analogue. The particularly strong effect of polymer **5** may arise from the combined contribution of cationic interactions and hydrogen-bonding motifs, which could strengthen polymer association with the *C. albicans* cell wall and thereby facilitate aggregation-driven biofilm development. Reference compounds show that none of the structural elements alone deliver the pronounced effects. Although the polymers did not exert pronounced antifungal activity against planktonic *Candida* cells, their marked effects on biofilm formation may still be relevant for biofilm-oriented antimicrobial diagnostic strategies. Combination approaches that target complementary aspects of biofilm-associated tolerance are also increasingly discussed as promising strategies against fungal biofilm infections.^109^ In this context, polymers **4** and **5** as well as polymer **3** in the case of *C. albicans* should not be regarded as stand-alone antifungal agents. Rather, they appear to function as biofilm-modulating materials that influence adhesion, aggregation, or surface colonization without affecting the overall cell viability. By shifting fungal populations from a planktonic to a biofilm-associated phenotype, these polymers may provide opportunities for diagnostic applications and could potentially facilitate the target delivery or efficacy of established antifungal treatments.^110^ Studies show that antimicrobial polymers and cationic materials can interact with fungal membranes or cell surfaces and can be combined with conventional antimicrobial agents to enhance activity. Moreover, quaternary ammonium compounds have been reported to display structure-dependent antiadhesive, antibiofilm, or fungicidal effects against yeasts.^109^ Combination-based approaches are particularly relevant because QACs and azole antifungals have shown synergistic activity in other antimicrobial settings.^109^ Nevertheless, the present study did not test such combinations, and the potential use of these polymers together with standard biocides or antifungals should be considered a hypothesis that will be addressed in the future work.

## Conclusions

The combined information from NMR, elemental analysis, and TGA allows for the estimation that the DoF for the polymers with cationic side groups is between 91 and 100%. By investigating the chemical modification of the polymer PVBC, the polymer-analogous reaction with azaadamantan-4-one **1** to obtain **3** shows the highest DoF followed by azaadamantane **2** and trimethylamine to afford **4** and **5**. The synthesized polyQACs—particularly polymer **5**—exhibit pronounced and species-dependent effects on biofilm formation by *C. auris*, *C. parapsilosis*, and *C. albicans*, ranging from complete inhibition to significant promotion, with remarkably high values of up to 2600% achieved depending on the *Candida* species and polyQAC structure. The systematic variation of the polymers leads to the proposal of a tentative mechanistic explanation of the structure–property relationship. This shows the necessity of synergistic interactions of various binding motifs between the polymers and *Candida* cell surfaces. Such behavior could be very attractive for the development of new (biofilm) diagnostic applications. Furthermore, the strong and species-dependent modulation of biofilm formation as well as the fact that cell viability seems to be largely unaffected highlights the potential of these polymers as experimental tools for studying fungal adhesion, aggregation, and biofilm development.

## Supplementary Material



## Notes

Viability and biofilm tests with various concentrations and determination of adsorbed amounts of polymers DSC and TGA (DOCX)
