# Supplementary material for: Contrasting Biofilm-Modulating Effects of Polymeric Quaternary Ammonium Compounds on the Pathogenic Yeasts Candida albicans, Candidozyma auris, and Candida parapsilosis
Source: ACS Omega. 2026 Jul 10;11(28):42061–75. doi: 10.1021/acsomega.6c02434 (PMC13393035; doi:10.1021/acsomega.6c02434)
Supplement: Supplementary file 1 [file ao6c02434_si_001.docx]

*Supporting Information*

Contrasting Biofilm Modulating Effects of Polymeric Quaternary Ammonium Compounds on the Pathogenic Yeasts *Candida albicans*, *Candidozyma auris* and *Candida parapsilosis*

Andreas Schelhorn,^†,∥^ Josef Achhammer,^†^ Denis Hirsch,^†^ Jan-Christoph Walter,^‡,§,∥^ Daniel Gruber,^‡,§^ Ann-Kathrin Kissmann,^‡,§^ Frank Rosenau,^‡^ and Ulrich Ziener^†,*^

† Institute of Organic Chemistry III-Macromolecular Chemistry and Organic Materials, University of Ulm, Albert-Einstein-Allee 11, D-89081 Ulm, Germany

‡ Institute of Pharmaceutical Biotechnology, Ulm University, Albert-Einstein-Allee 11, 89081 Ulm, Germany

§ Faculty of Medicine and Dentistry, Danube Private University, Steiner Landstraße 124, 3500 Krems an der Donau, Austria

^∥^A.S. and J.-C.W. contributed equally to this work.

*Email: [ulrich.ziener@uni-ulm.de](mailto:ulrich.ziener@uni-ulm.de)

**Results**


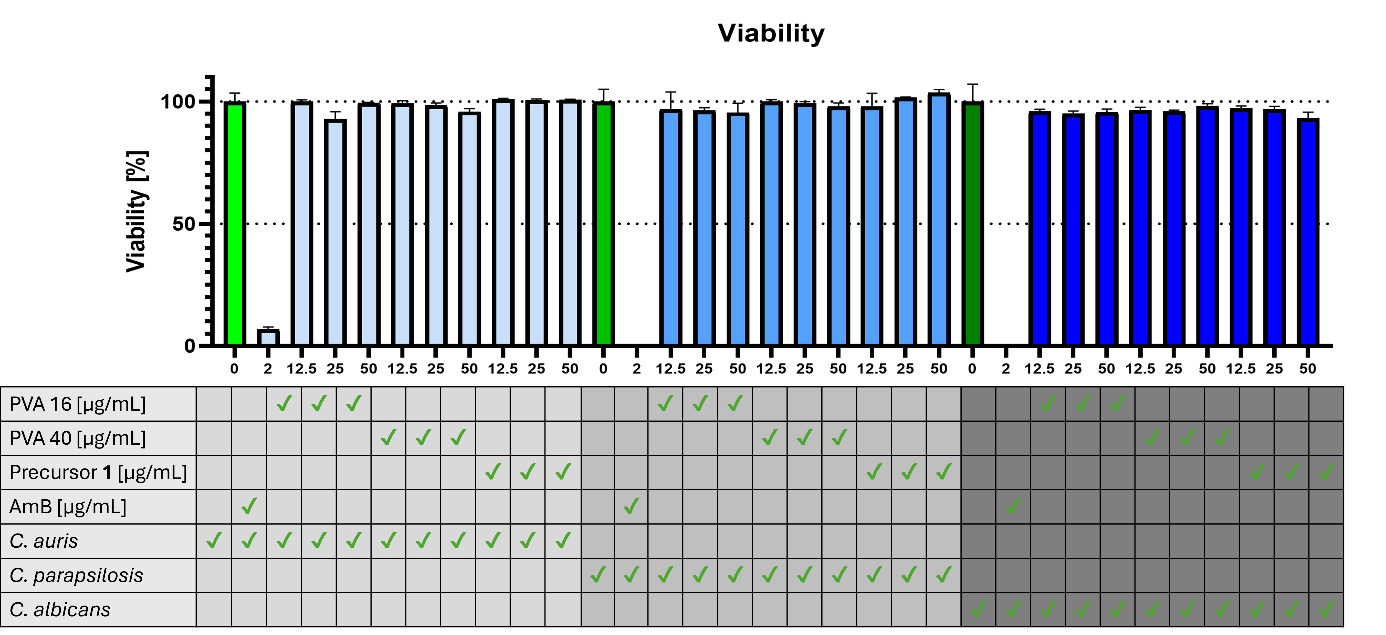


**Figure S 1.** Viability of C. auris, C. parapsilosis and C. albicans cells incubated in 96-well-plates with reference compounds (blue) at different concentrations and 2 µg/mL of Amphotericin B as a control. Untreated cells (green) serve as a reference and are set to 100% cell viability.


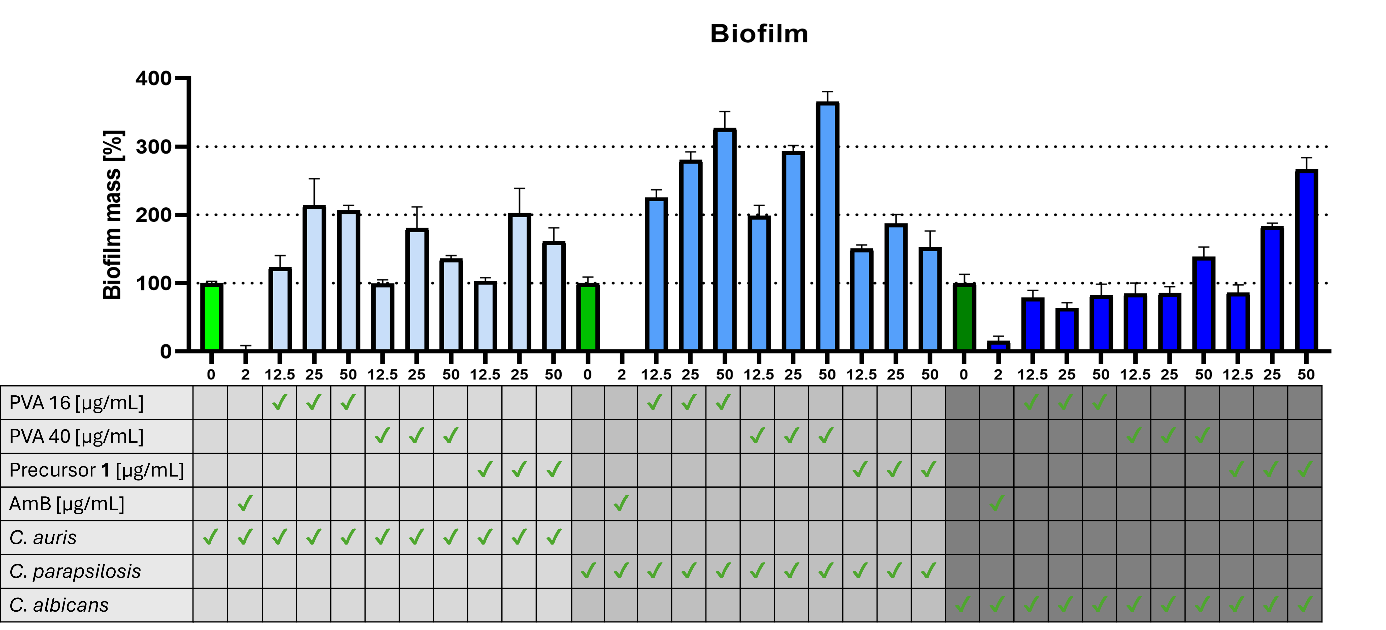


**Figure S 2.** Biofilm quantification of C. auris, C. parapsilosis and C. albicans cells incubated in 96-well-plates with reference compounds (blue) at different concentrations and 2 µg/mL of Amphotericin B as a control. Untreated cells (green) serve as a reference and are set to 100% biofilm mass.


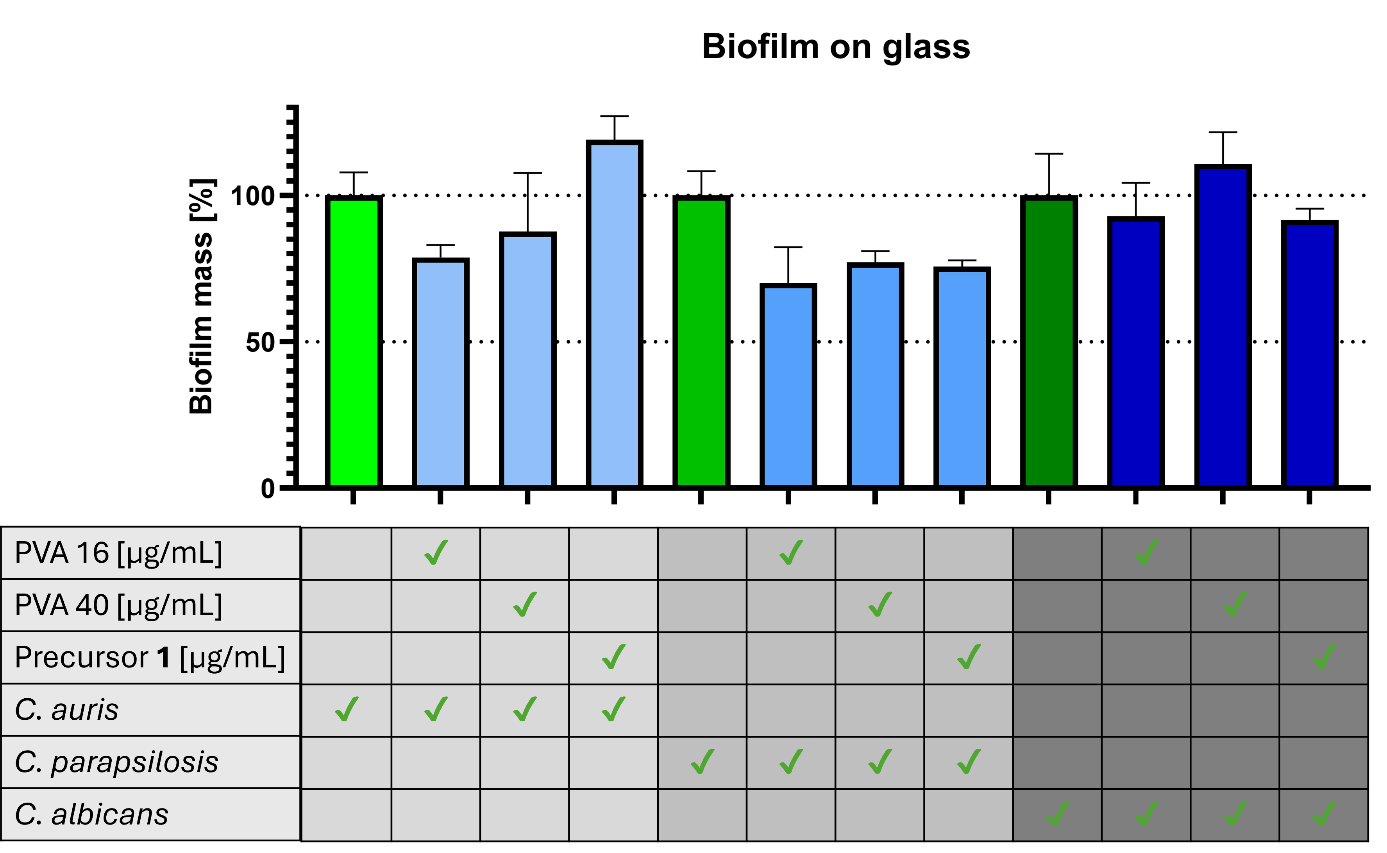


**Figure S 3.** Viability of C. auris, C. parapsilosis and C. albicans cells incubated in 96-well-plates with reference compounds at different concentrations and 2 µg/mL of Amphotericin B as a control. Untreated cells (green) serve as a reference and are set to 100% cell viability.

**Estimation of adsorbed amounts of polymers on glass**

To estimate the adsorbed amounts of polymers **3**, **4** or **5** on the HPLC glass vials, silicon dioxide particles (silica gel 60 M, 0.04–0.063 µm, Macherey Nagel, Germany) were selected as the reference surface. 1.5 mL of the polymer solution in water (**3**, **4**: 0.12 mg ml^-1^, **5**: 0.10 mg ml^-1^) were incubated with different amounts of dry silica gel for 24 h and centrifuged. The supernatant was examined using UV/Vis spectroscopy and the concentration of the polymer was determined. The amount of adsorbed polymer was calculated from the difference to the solutions without silicon dioxide particles.


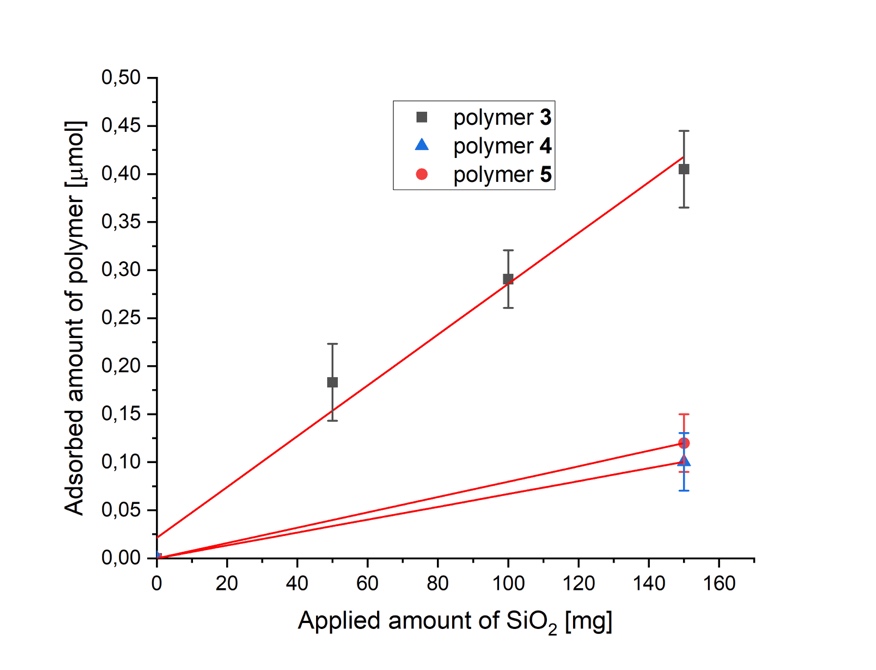


**Figure S 4.** Adsorbed amount of polymer on various amounts of silica particles. The data for polymer **3** display a reasonable linear dependence why for polymers **4** and **5** only one data point was measured.

Since the pores of silicon dioxide are too small to be occupied by the polymer molecules to any significant extent, an accessible surface area of 0.05 m² g⁻¹ was assumed. On this basis, the following quantities adsorbed to the glass vial (surface area 1.3 cm²) could be calculated (Table S1).

**Table S 1.** Estimated adsorbed amounts of polymer on the glass vial surfaces.

|  | Mass per vial/μg |
| --- | --- |
| Polymer **3** | 1.6$\pm$0.1 |
| Polymer **4** | 1.2$\pm$0.1 |
| Polymer **5** | 0.6$\pm$0.1 |

**TGA**


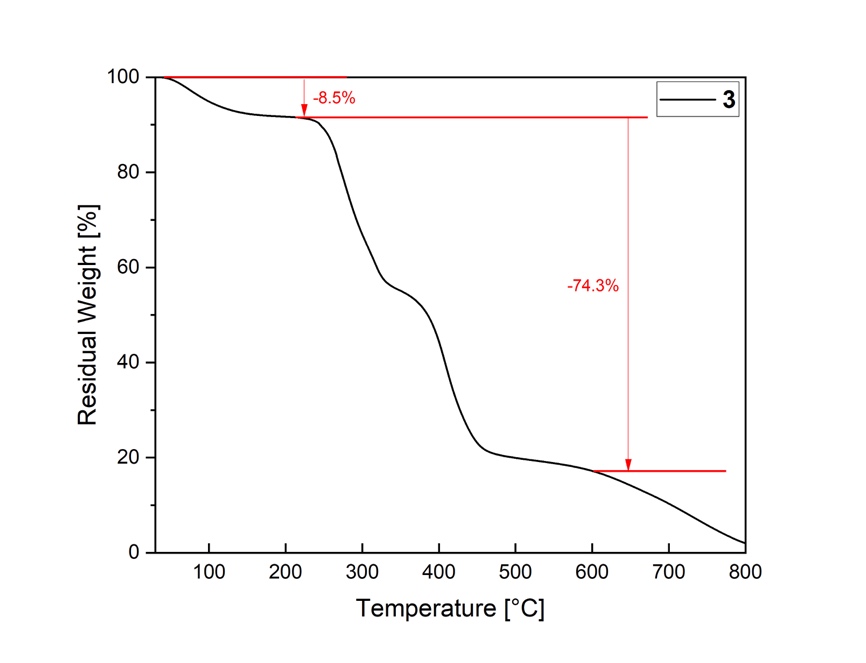


**Figure S 5**. TGA of **3** (heating rate: 10 ºC min^-1^ under nitrogen flow (20.0 mL min^-1^) from room temperature to 800 °C).


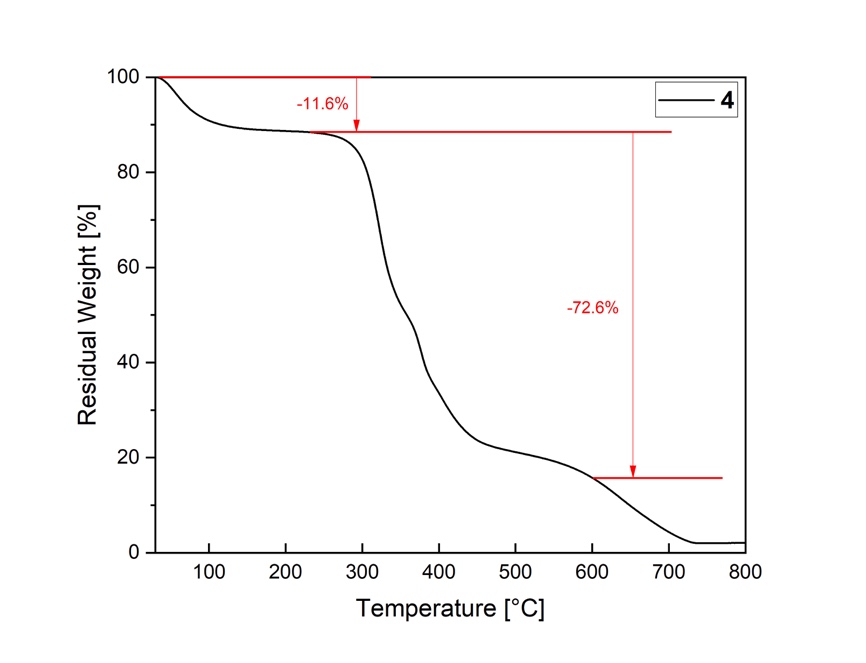


**Figure S 6**. TGA of **4** (heating rate: 10 ºC min^-1^ under nitrogen flow (20.0 mL min^-1^) from room temperature to 800 °C).


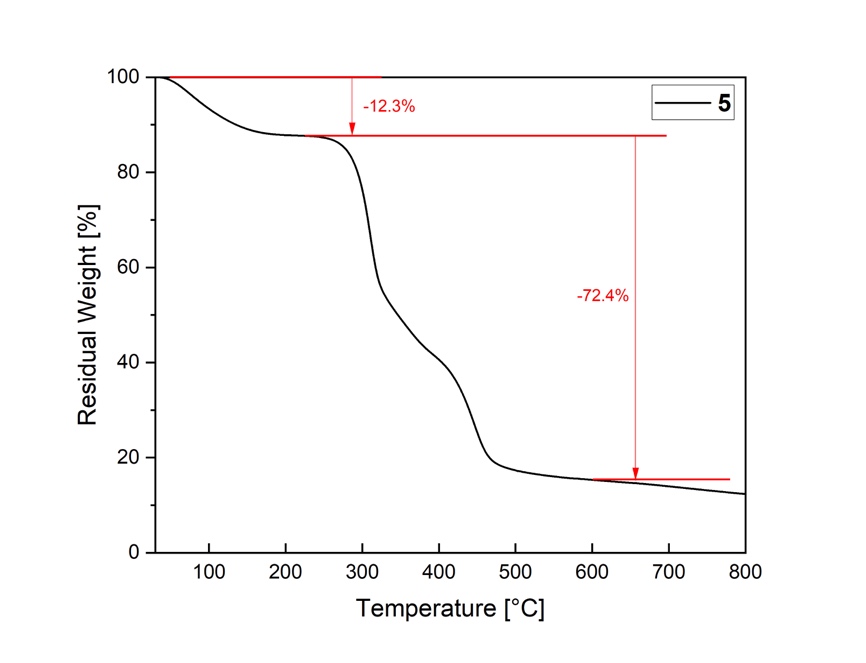


**Figure S 7**. TGA of **5** (heating rate: 10 ºC min^-1^ under nitrogen flow (20.0 mL min^-1^) from room temperature to 800 °C).

For the determination of the DoF values, it is assumed that the quaternary ammonium groups decompose completely up to 600 °C, whereas the remaining PVBC backbone undergoes degradation of only about 65% at this temperature.^1^

$$\frac{M_{a}+0.65*M_{b}}{M_{P}}*x+\left( 1-x \right)*0.65={\Delta m}_{exp}$$

with *M*_a_: molar mass amine, *M*_b_: molar mass vinylbenzyl chloride, *M*_P_: molar mass polymer, *x*: DoF, and *Δm*_exp_: experimental mass loss at 600 °C with respect to the mass at ca. 250 °C.

This yields DoF values of 1.65, 1.03, and 1.01 for polymers **3**, **4**, and **5**, respectively. The value significantly exceeding unity observed for **3** suggests that the presence of trimethylamine promotes additional degradation of the PVBC backbone beyond the assumed 65%.

**DSC**


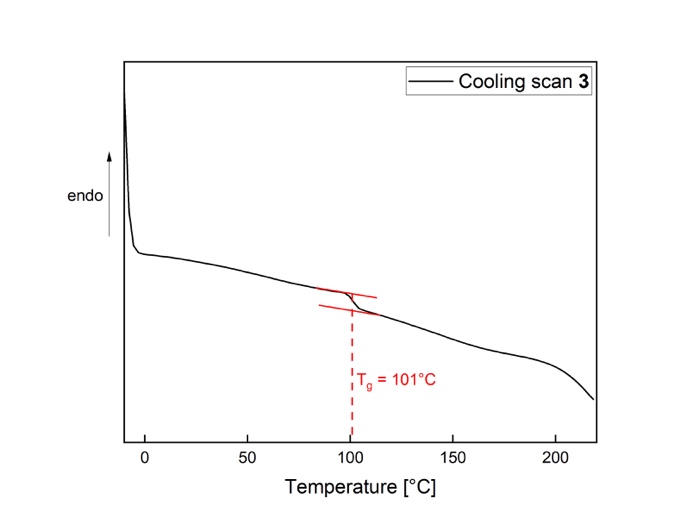


**Figure S 8**. DSC of **3** (heating/cooling rate of 10 °C min^-1^ for three scanning cycles of heating-cooling in N_2_ atmosphere).


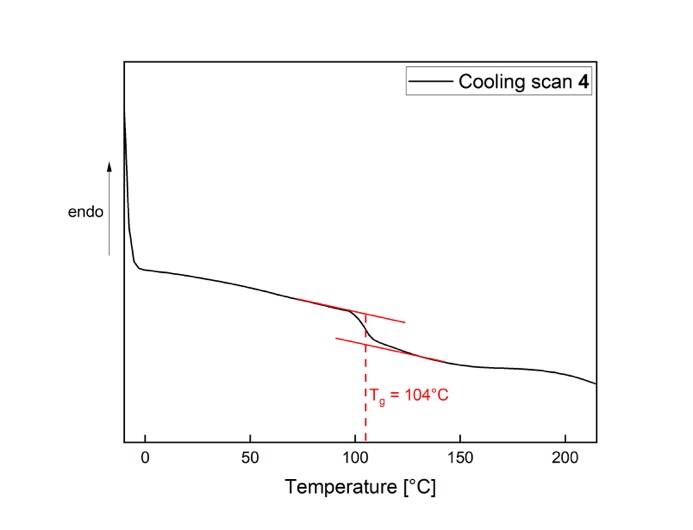


**Figure S 9**. DSC of **4** (heating/cooling rate of 10 °C min^-1^ for three scanning cycles of heating-cooling in N_2_ atmosphere).


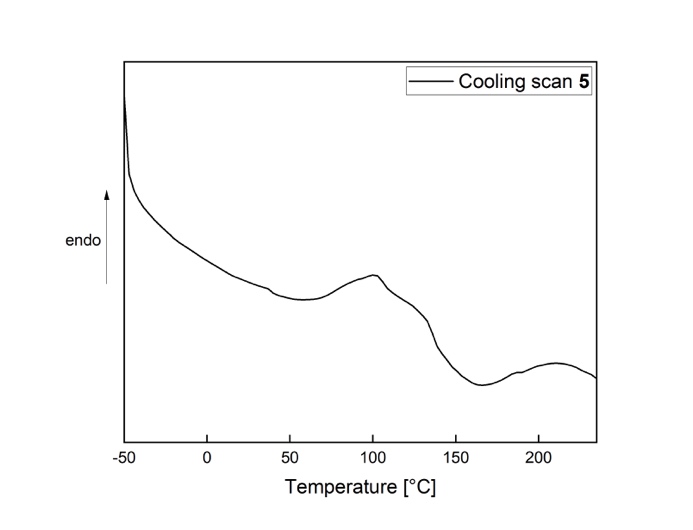


**Figure S 10**. DSC of **5** (heating/cooling rate of 10 °C min^-1^ for three scanning cycles of heating-cooling in N_2_ atmosphere).

**References**

(1) Mathew, M. E.; Ahmad, I.; Thomas, S.; Kassim, M.; Daik, R. A Preliminary Study on the Synthesis of poly(vinylbenzyl chloride) with Different Solvents. *Sains Malays.* **2021**, *50* (6), 1767-1773. DOI: 10.17576/jsm-2021-5006-22
